# A novel protocol for the one-pot borylation/Suzuki reaction provides easy access to hinge-binding groups for kinase inhibitors†
†Electronic supplementary information (ESI) available. See DOI: 10.1039/c5ob01915j



**DOI:** 10.1039/c5ob01915j

**Published:** 2015-12-01

**Authors:** A. Hooper, A. Zambon, C. J. Springer

**Affiliations:** a The Institute of Cancer Research , Cancer Research UK Cancer Therapeutics Unit , Division of Cancer Therapeutics , 15 Cotswold Road , Sutton , Surrey SM2 5NG , UK . Email: Caroline.Springer@icr.ac.uk

## Abstract

The one-pot borylation/Suzuki reaction is a very efficient means of accessing cross-coupling products of two aryl-halide partners that generally requires the use of specific catalysts or ligands and/or relatively long reaction times. This new microwave-assisted method provides a quick one-pot borylation/Suzuki reaction protocol that we applied to the synthesis of various bi- or poly-aryl scaffolds, including a variety of aryl and heteroaryl ring systems and the core frameworks of kinase inhibitors vemurafenib and GDC-0879.

## Abstract 2

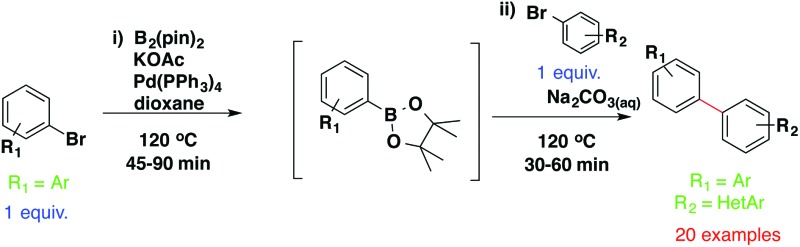
This new microwave-assisted method provides a quick one-pot borylation/Suzuki protocol that does not require additional ligands nor double loading of catalyst.

## Introduction

Kinases are key functional proteins that regulate signal transduction pathways in cells by catalyzing phosphorylation of serine, threonine, or tyrosine residues. Deregulation of protein kinases is implicated in many diseases including cancer, diabetes, and inflammation: as a consequence kinases have been major targets for recent small molecule drug development.^1–3^

A common feature of most kinase inhibitors is the presence of a hinge-binding moiety,^4^ a group that is able to form hydrogen bonds to the cleft between N- and C-lobes of the kinase known as the hinge region.^4^ Typically, a hinge-binding structure consists of a hetero-aromatic group containing hydrogen bond donors and/or acceptors in either a mono- or bi-dentate fashion. This mode of binding mimics that of the adenosine ring of the natural kinase ligand, ATP.

The palladium-catalysed Suzuki–Miyaura reaction, which couples aryl halide and aryl boronic species for the formation of new C–C bonds,^5–8^ is particularly suited to access hinge binding fragments thanks to its tolerance of functional groups and mild reaction conditions. Furthermore, extensive literature describes a wide range of experimental procedures.^9,10^

Despite its wide scope, the Suzuki–Miyaura cross-coupling reaction has a number of limitations such as lack of availability, high expense and instability of certain boronic species. In order to circumvent these issues, Miyaura explored the use of bis(pinocolato)diboron as the boronic acid equivalent in a one-pot borylation/Suzuki reaction, which eliminates the need to isolate the boronic intermediate. Despite subsequent improvements to the methodology,^11–15^ current one-pot borylation/Suzuki protocols require double loading of specific catalysts, use of additional ligands or relatively long reaction times, and their scope is generally limited to one specific scaffold.^16–18^

Our aim was to develop a robust one-pot borylation/Suzuki protocol that employs one single loading of catalyst with no need for additional ligands and to use it to access a small panel of putative hinge binding fragments, which were then profiled for kinase selectivity.

## Results and discussion

As a model reaction for the optimisation of the one-pot protocol, we selected the coupling of 5-bromoindanone **1a** and 3-bromopyridine **3a** to give 3-pyridinylindenone **4a**, mediated by the formation of pinacolate boronic ester **2a**. We reasoned that the structure of **4a** could act as a basic scaffold for a kinase inhibitor, with the pyridine group acting as hinge binder and the indanone elaborating into the ATP binding pocket. Variation of this basic structure would then allow us to access a panel of compounds with potential kinase activity.

The original borylation conditions, developed by Miyaura^11^ were assessed utilising Pd(dppf)Cl_2_ as the catalytic species and KOAc as the base. Although this yields the boronic ester **2a** with 100% conversion, addition of 3-bromopyridine **3a**, along with catalyst and base did not yield **4a** (Table 1, entry 1). Pd(PPh_3_)_4_ as catalyst was assessed for the Suzuki step but again **4a** was not formed (entry 2). However, the borylation reaction time could be shortened to 1 hour at 120 °C under microwave irradiation resulting in the formation of intermediate **2a**. This is a significant time reduction from 18 h needed at 80 °C using both catalytic species (entries 3 and 4). From this point, only tetrakis(triphenylphosphine)palladium(0) as catalyst was utilized to avoid a mixture of catalytic species as this was deemed most suitable due to its wide use and cheaper cost.

**Table 1:** Optimisation of the reaction conditions^*a*^

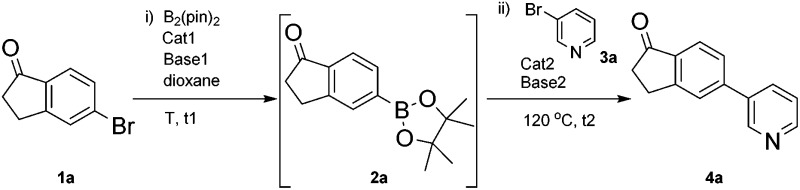							
Entry	Cat1/base1	*t*2 (h)	*T* (°C)	**2a** ^*b*^ (%)	Cat2/Base2	*t*2 (h)	**4a** ^*b*^ (%)
1	Pd(dppf)Cl_2_/KOAc	18	80	100	Pd(dppf)Cl_2_/KOAc	1	0
2	Pd(dppf)Cl_2_/KOAc	18	80	100	Pd(PPh_3_)_4_/KOAc	1	0
3	Pd(dppf)Cl_2_/KOAc	1	120^*c*^	100	Pd(PPh_3_)_4_/KOAc	1	0
4	Pd(PPh_3_)_4_/KOAc	1	120^*c*^ ^,^ ^*d*^	100	Pd(PPh_3_)_4_/KOAc	1	0
5	Pd(PPh_3_)_4_/KOAc	1	120^*c*^	100	Pd(PPh_3_)_4_/Na_2_CO_3_(aq)	1	100
6	Pd(PPh_3_)_4_/Na_2_CO_3_(aq)	1	120^*c*^	0^*e*^	—	—	—
7	Pd(PPh_3_)_4_/KOAc	45 min	120^*c*^	100	—/Na_2_CO_3_(aq)	30 min	100

^*a*^Reaction conditions: **1a** (1 equiv.), B_2_(pin)_2_ (1.2 equiv.), catalyst (10 mol%) and base (3 equiv.) in dioxane (0.5 M) followed by **3a** (1 equiv.), catalyst (10 mol%) and base (2 equiv.).

^*b*^Conversion by LCMS.

^*c*^Reactions performed in a microwave.

^*d*^Initially heated to 80 °C over 18 h but after no product **2a** formation was observed, it was heated to 120 °C in a microwave for 1 h.

^*e*^Only indanone dimer observed.

**Fig. 1:**
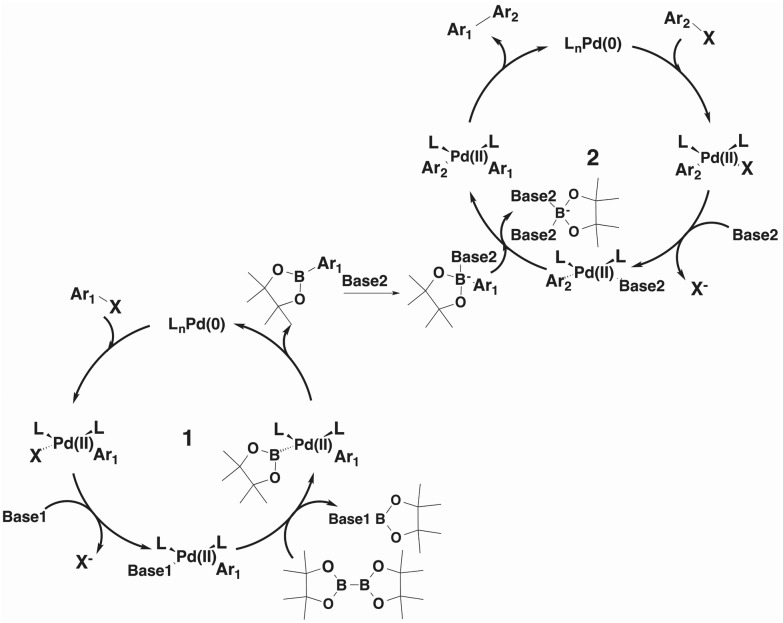
Proposed catalytic cycle.

To identify the best reaction conditions to access **4a** for a one pot reaction, we postulated a bicyclic mechanism merging the catalytic cycles **1** and **2** elucidated by Miyaura and Suzuki for the borylation and coupling steps respectively,^11,19^ as outlined in Fig. 1. Cycle **1** is a typical borylation cycle with oxidative–addition of the first halide to the catalytic species, oxidising the palladium species from the neutral state of the Pd(PPh_3_)_4_ to the Pd(ii) species. Base1 then displaces the halide within the catalytic complex, activating it for the subsequent transmetallation step with bis(pinocolato)diboron.^11,19^ A final reductive–elimination step recovers the Pd(0) catalytic species and completes cycle **1**, releasing the aryl-boronic species; at this stage the reactions carried out in entries 1–4 stalled.

Similarly, catalytic cycle **2** is initiated by oxidative addition of the second halide to the catalyst and the displacement of the halide by Base2 followed by addition of the aryl boronate to form the biaryl-substituted palladium species. Complexation of a base to the aryl boronate is essential at this stage in order to accelerate the transmetallation step^20^ by forming a more reactive boronate to interact with the palladium species^21^ and facilitate an intramolecular transmetallation.^22^ We postulated that a stronger base is required at this step in cycle **2** than the one needed in cycle **1**, which only has the role to displace the halide from the first palladium species.

We then applied a change of base to our model system, introducing sodium carbonate as second base after the formation of intermediate **2a**, and pleasingly the pyridinylindenone product **4a** is formed with 100% conversion (entry 5). As a further confirmation of the reaction mechanism, the introduction of sodium carbonate in the borylation step affords only the homocoupling product (entry 6).

Once the optimal base/catalyst system was identified, we worked to eliminate the second catalyst loading and decrease the reaction times. In these optimised conditions, 100% conversion to product **4a** was obtained (entry 7) using a single initial loading of Pd(PPh_3_)_4_, with 45 minutes at 120 °C under microwave irradiation for the borylation step, followed by addition of **3a** and Na_2_CO_3_ with further heating to 120 °C for 30 min in the Suzuki step.

Next, we applied this optimised protocol to the synthesis of a small panel of putative kinase inhibitors. To explore the scope of the reaction and provide a set of hinge-binding fragments, we included in the reaction panel keto, boc-protected aniline, halo, aryl, indanone, pyridyl, pyrazole, azaindole and quinoline functional groups. To this end, a selection of eight commercially available, relatively undecorated halides was chosen: four heteroaromatic rings containing either a hydrogen bond acceptor, a hydrogen bond donor or both (**3a–d**) and four phenyl moieties (**1a–d**), to be coupled in combination to obtain 16 products (**4a–p**). Within this set is compound **4j**, which is a precursor of the BRAF-selective inhibitor, vemurafenib. The isolated yields obtained were good to excellent with the single exception of **4b** (Table 2). Pleasingly, regioselectivity in the coupling reaction was obtained for **1c** (entries 9 to 12) and **3d** (entries 4, 8, 12 and 6), which only reacted at the bromine and not at the chlorine.

**Table 2:** Scope of reaction to perform basic scaffolds^*a*^

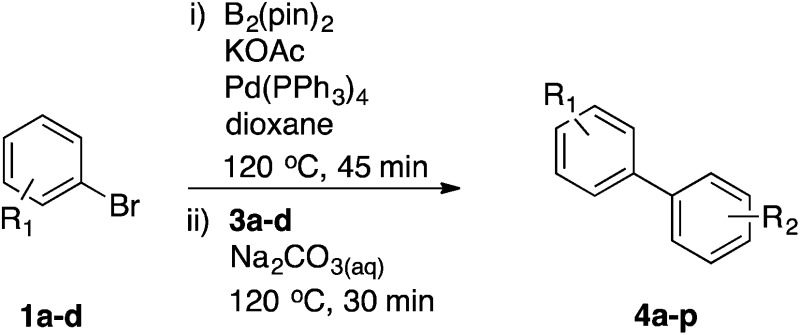				
Entry	First halide	Second halide	Product	Yield^*b*^ (%)
1	**1a**	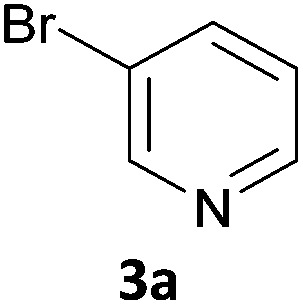	**4a**	70
2	**1a**	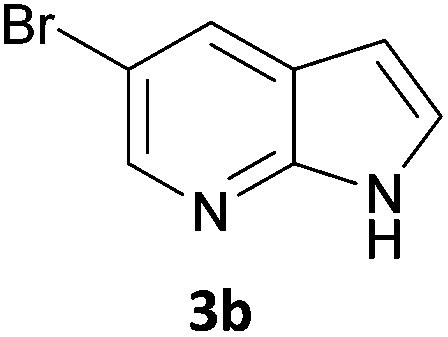	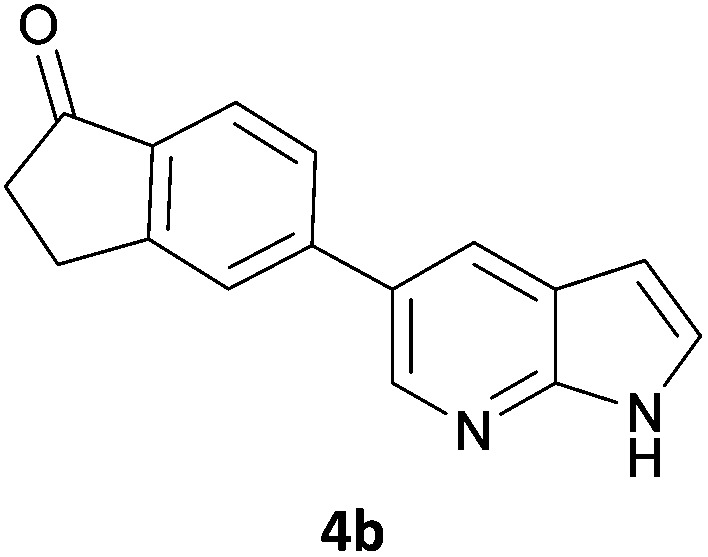	12
3	**1a**	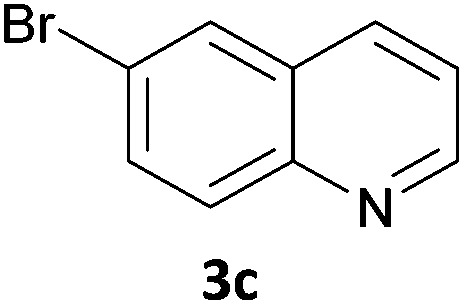	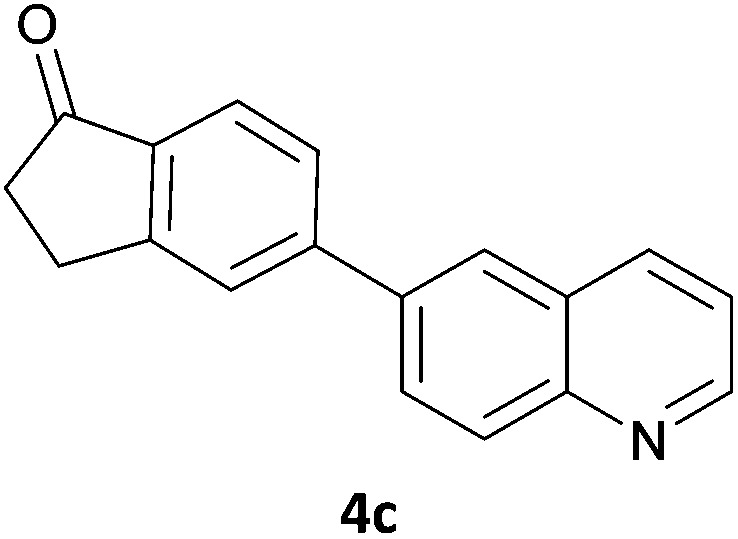	67
4	**1a**	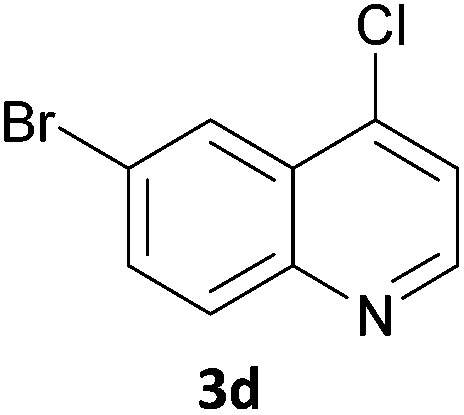	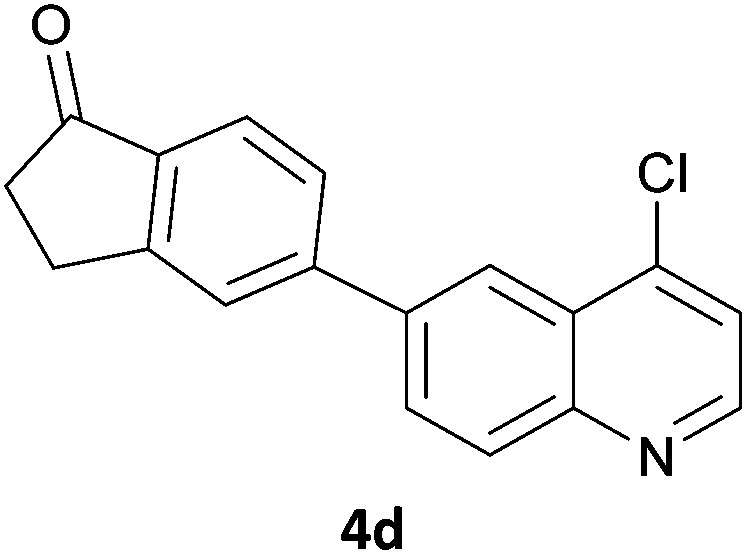	100
5	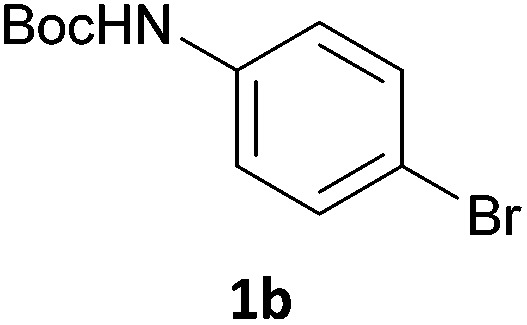	**3a**	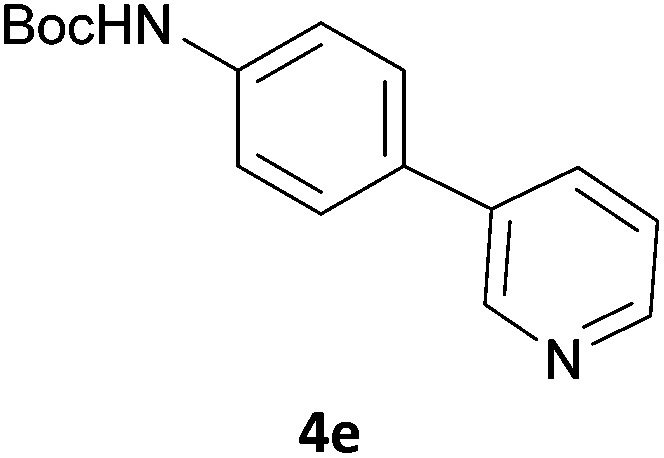	52
6	**1b**	**3b**	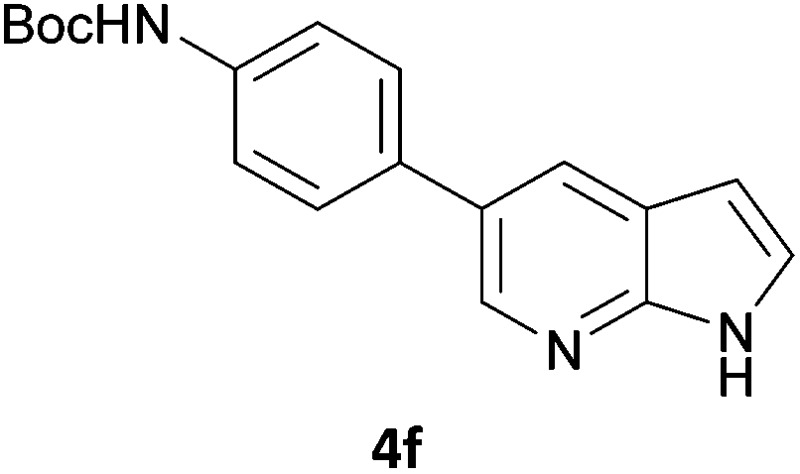	53
7	**1b**	**3c**	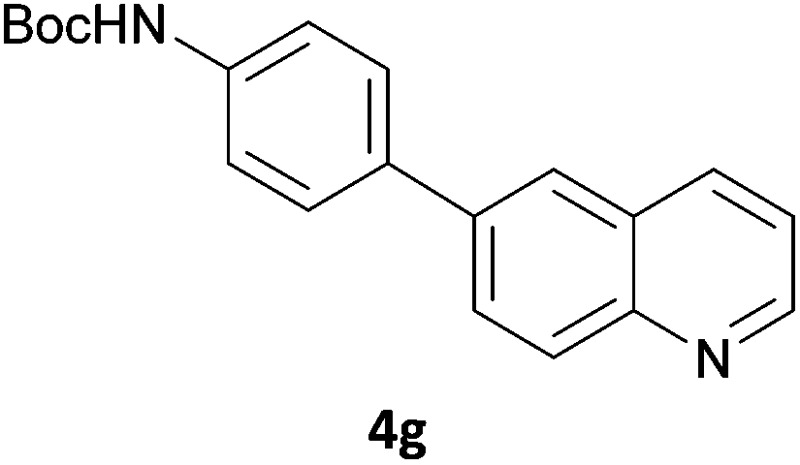	46
8	**1b**	**3d**	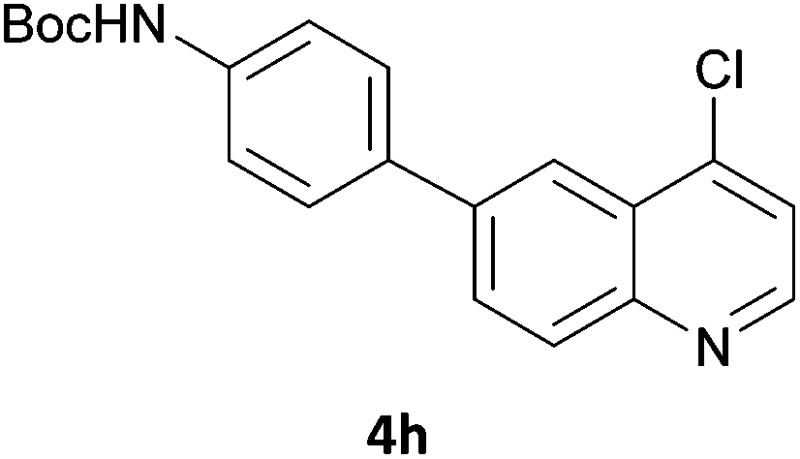	40
9	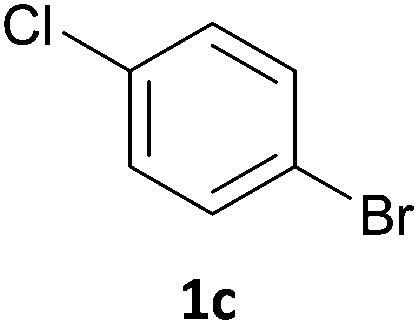	**3a**	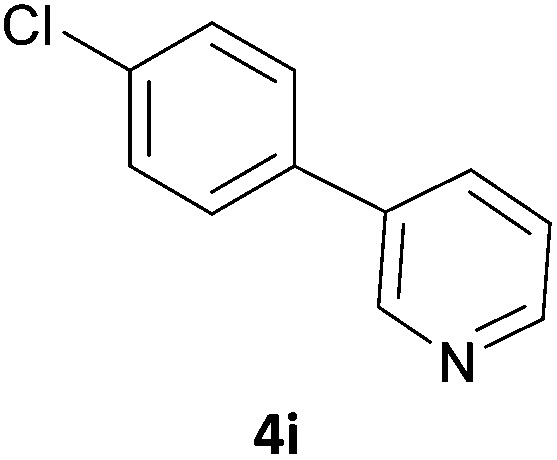	42
10	**1c**	**3b**	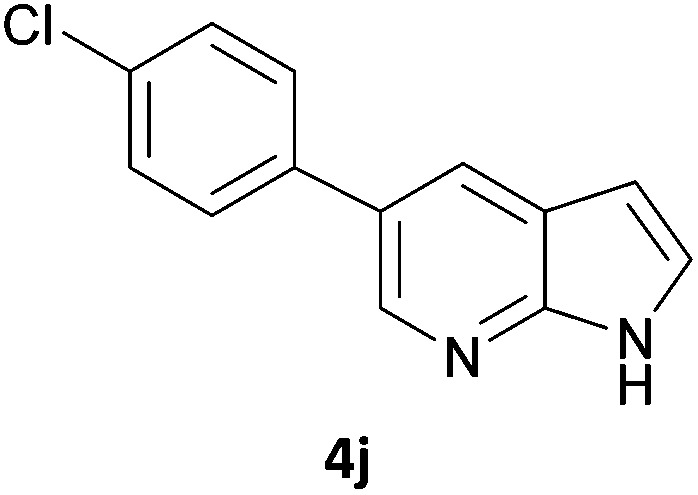	32
11	**1c**	**3c**	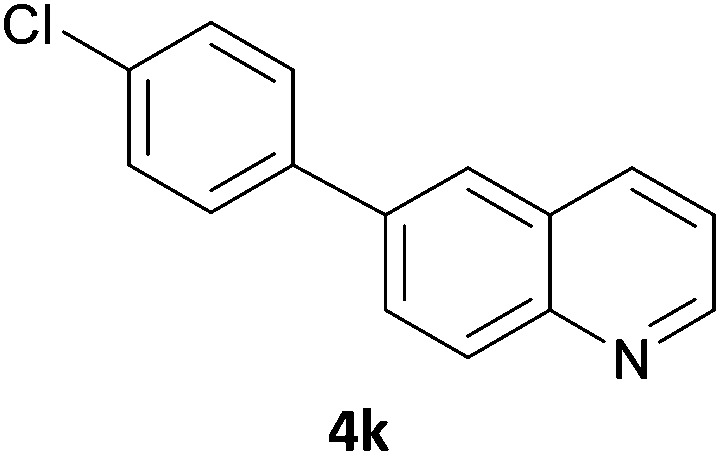	62
12	**1c**	**3d**	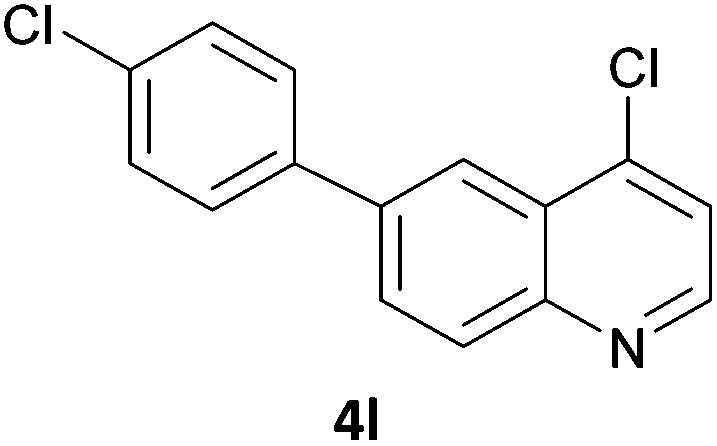	49
13	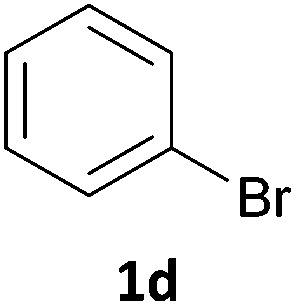	**3a**	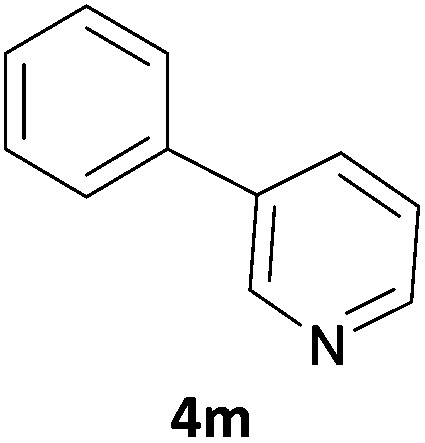	81
14	**1d**	**3b**	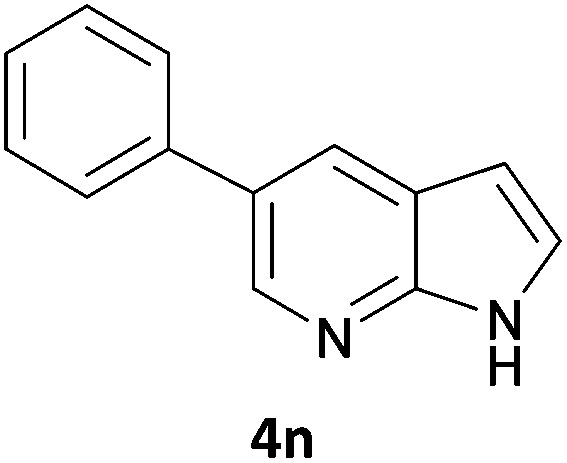	45
15	**1d**	**3c**	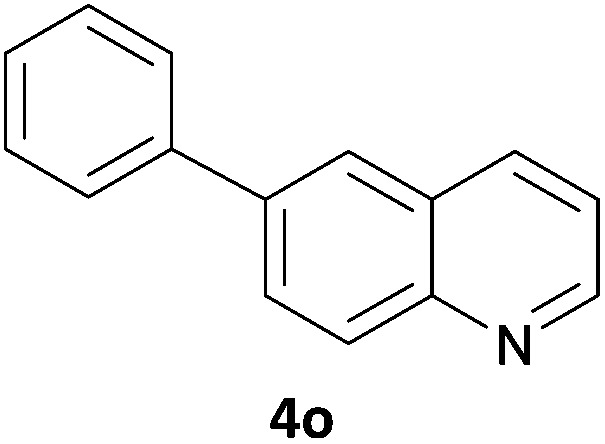	87
16	**1d**	**3d**	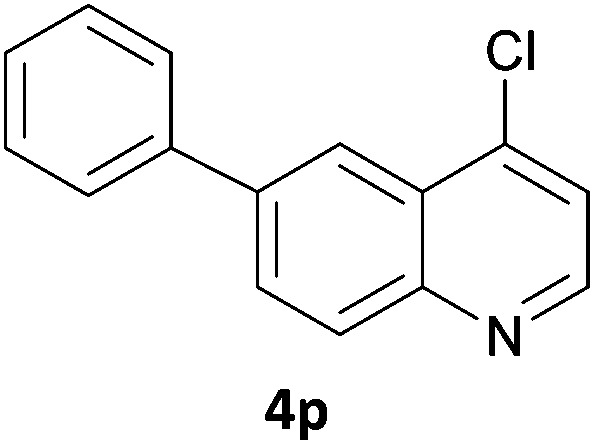	68

^*a*^Reaction conditions: first halide (1 equiv.), B_2_(pin)_2_ (1.2 equiv.), Pd(PPh_3_)_4_ (10 mol%) and KOAc (3 equiv.) in dioxane (0.5 M) followed by second halide (1 equiv.) and 2 M Na_2_CO_3_ (aq) (2 equiv.).

^*b*^Isolated yield.

The new protocol was then evaluated for the synthesis of more complex scaffolds; coupling of halides **1a–d** with the trityl-protected 4-(4-bromopyrazolyl)pyridine **3e** provided GDC-0879^23^ analogues **5a–d** in moderate to good yields (Table 3) with a slightly modified protocol allowing for longer reaction times.

**Table 3:** Scope of reaction to form complex scaffolds^*a*^

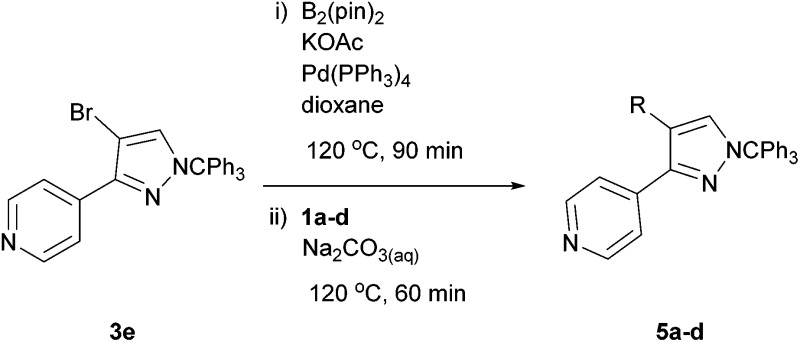				
Entry	First halide	Second halide	Product	Yield^*b*^ (%)
1	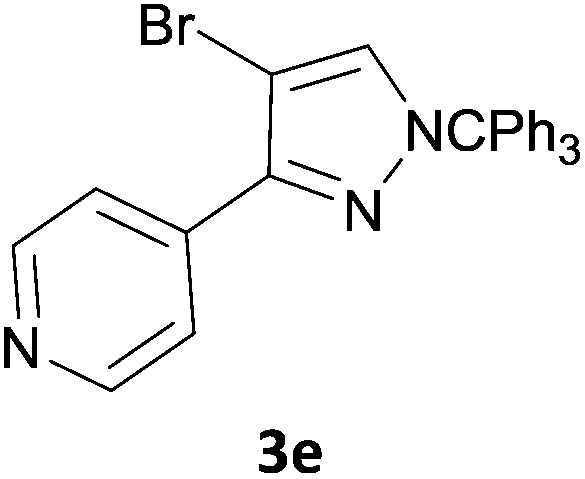	**1a**	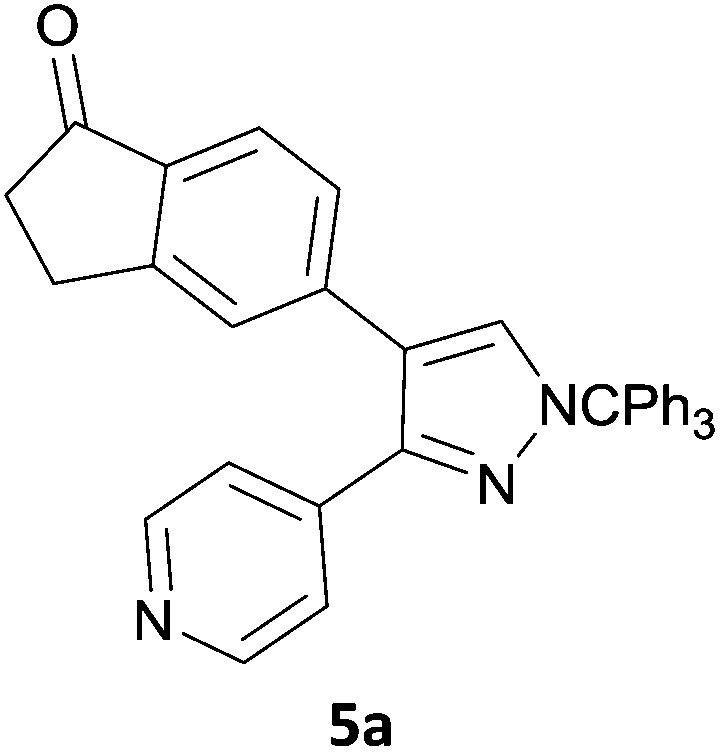	57
2	**3e**	**1b**	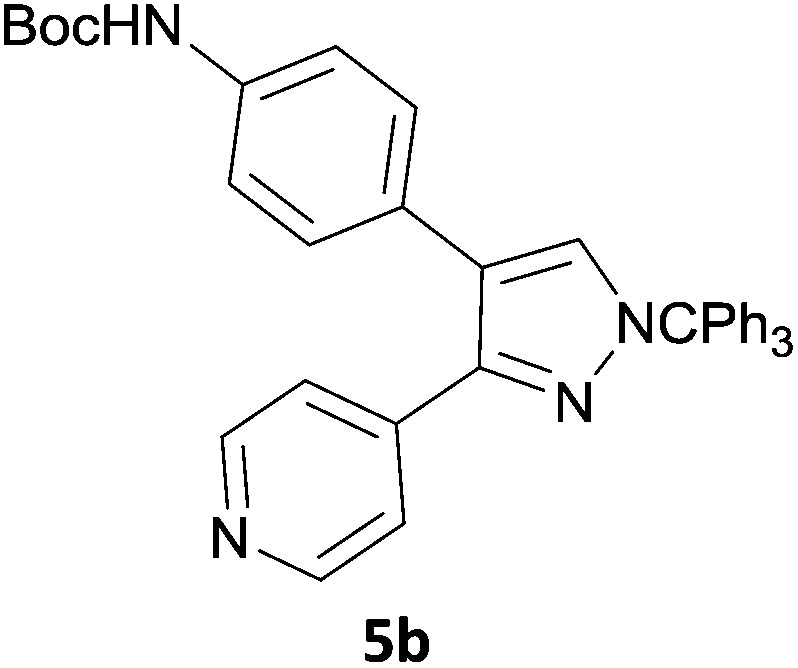	33
3	**3e**	**1c**	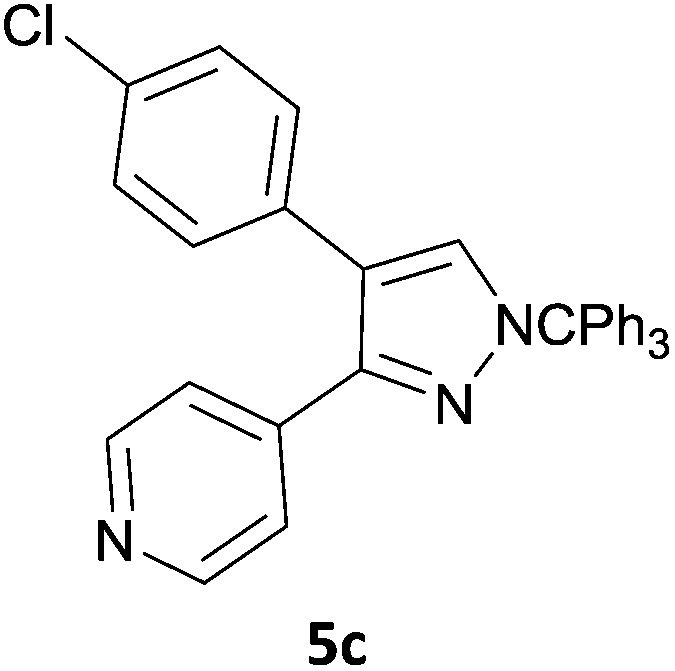	49
4	**3e**	**1d**	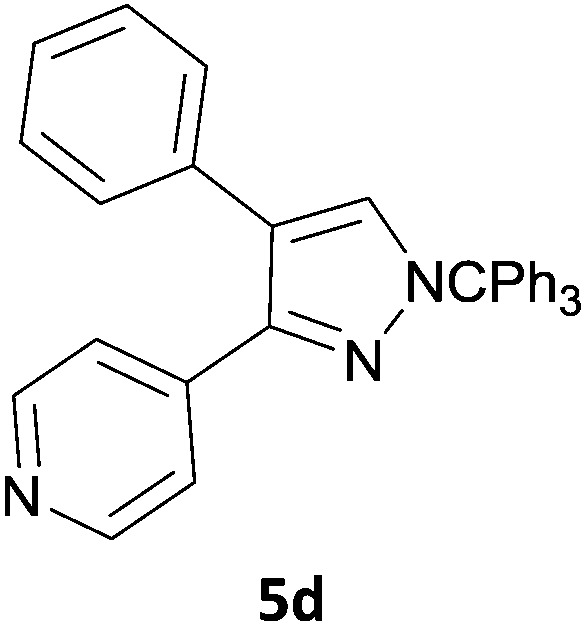	65

^*a*^Reaction conditions: first halide (1 equiv.), B_2_(pin)_2_ (1.2 equiv.), Pd(PPh_3_)_4_ (10 mol%) and KOAc (3 equiv.) in dioxane (0.4 M) followed by second halide (1 equiv.) and 2 M Na_2_CO_3_ (aq) (2 equiv.).

^*b*^Isolated yield.

The 20 compounds synthesised were assessed on a small panel of kinases using the ProfilerPro® Selectivity Assay Kits (Caliper Life Sciences, Inc.). The inhibition of 24 kinases was determined at a compound concentration of 300 μM; of the 20 compounds, 11 showed greater than 50% inhibition of at least one kinase (Table 4) with compounds **4j**, **4n**, **4o** and **4p** showing greater than 90% inhibition for at least one kinase. Of these, **4o** and **4p** have a selective profile, inhibiting just ABL (entry 10, **4o**) or both ABL and CK1 (entry 11, **4p**) at the concentrations used. There is one commercially available ABL inhibitor which contains a quinoline moiety, Rebastinib (DCC-2036), but this has a binding mode in which the quinolone does not interact with the hinge region.^24^

**Table 4:** Results of kinase screen using a small panel of kinase inhibitor-like scaffolds

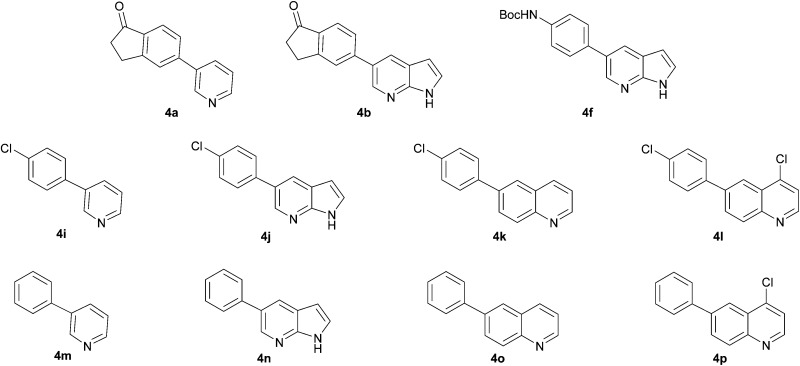																				
Entry	Compound	Kinase % inhibition																		
		AurA	RSK1	PRAK	Erk1	PKD2	CK1δ	CHK1	ABL	FYN	LYNα	CHK2	MET	LCK	SRC	GSK3β	Erk2	PKACα	INSR	MSK1
1	**4a**	51	41	19	14	38	38	10	41	–2	33	35	16	31	25	56	14	6	24	28
2	**4b**	76	–2	3	16	6	46	15	37	1	20	41	16	16	20	26	19	–14	61	4
3	**4f**	60	20	40	8	18	50	13	23	–25	24	35	9	29	8	8	–7	37	46	19
4	**4i**	61	52	26	7	32	34	0	68	54	63	25	17	61	43	30	10	52	24	42
5	**4j**	89	91	86	55	87	73	6	87	79	30	43	47	16	79	45	53	46	58	69
6	**4k**	45	31	22	6	34	70	–9	65	–1	29	18	29	43	19	26	1	22	21	13
7	**4l**	7	3	–1	0	–35	68	–7	36	18	–7	3	16	11	3	60	3	29	12	13
8	**4m**	51	30	27	8	13	58	–12	43	27	46	37	28	18	25	14	4	56	26	26
9	**4n**	99	70	47	62	92	94	80	85	53	76	91	52	63	62	57	66	61	93	43
10	**4o**	45	34	12	7	16	44	1	95	30	49	18	35	54	23	26	1	37	44	15
11	**4p**	66	48	14	6	18	92	–1	94	48	64	37	35	67	36	50	–7	59	49	24

In order to validate hits **4o** and **4p**, ABL IC_50_s were determined (Invitrogen) at a starting compound concentration of 100 μM. Compound **4o** inhibits ABL with an IC_50_ of 8 μM, corresponding to a high ligand efficiency of 0.45, as shown in Table 5 and, although it has a higher IC_50_, a similar ligand efficiency is observed for **4p** with ABL. Conversely, the IC_50_ of **4p** for CK1δ was >300 μM, further highlighting the selectivity of the scaffold.

**Table 5:** Determination of IC_50_ and ligand efficiency with ABL for **4o** and **4p** at compound concentration of 100 μM

Entry	Compound	ABL IC_50_ (μM)	Ligand efficiency
1	**4o**	8.11	0.45
2	**4p**	19.2	0.39

## Conclusions

In conclusion, a novel, quick and robust microwave-assisted protocol for the one-pot borylation/Suzuki reaction has been developed to access a small panel of putative kinase inhibitors containing a variety of aryl and heteroaryl ring systems. These scaffolds have been assessed for inhibition of a panel of kinases, and a ligand efficiency of 0.45 is observed with the kinase ABL for compound **4o**.
