# Supplementary material for: A novel protocol for the one-pot borylation/Suzuki reaction provides easy access to hinge-binding groups for kinase inhibitors
Source: Org Biomol Chem. 2015 Dec 1;14(3):963–9. doi: 10.1039/c5ob01915j (PMC4718143; doi:10.1039/c5ob01915j)
Supplement: Supplementary file 1 [file OB-014-C5OB01915J-s001.pdf]

Electronic Supplementary Material (ESI) for Organic & Biomolecular Chemistry.
This journal is © The Royal Society of Chemistry 2015

Supplementary Information

A novel protocol for the one-pot borylation/Suzuki reaction

A. Hooper,[a] A. Zambon[a+] and C. J. Springer[a+*]

Cancer Research UK Cancer Therapeutics Unit, Division of Cancer Therapeutics,
The Institute of Cancer Research, Haddow Laboratories,
Sutton, Surrey, SM2 5NG, UK.

1. Experimental S2
- **1.1 Kinase Assay S2**
- **1.2. General Experiment Details S2**
- **1.3. General procedure for synthesis of small panel of kinase-like scaffolds 4a-4p S3**
- **1.4. General procedure for synthesis of small panel of kinase-like scaffolds 5a-5d S8**

2. NMR Spectra S11
- **2.1 ^{1}H NMR S11**
- **2.2 ^{1}H NMR S21**

1. Experimental

1.1. Kinase Assay

IC_{50} determinations performed by the SelectScreen® Biochemical Kinase Profiling Service (Invitrogen). To test the enzyme selectively of the inhibitors, ProfilerPro kits (Caliper Life Sciences, Inc.) were used as described in the protocol.

1.2. General Experimental Details

Unless otherwise stated, reagents and solvents were purchased from commercial suppliers (Acros, Apollo, Fisher, Fluorochem, Sigma-Aldrich, Strem Chemicals Inc. and VWR) and used without further purification. Chromatography solvents were HPLC grade and were used without further purification. All reactions were carried out in oven-dried flasks under a positive pressure of Argon, and air and moisture sensitive reagents transferred *via* syringe. Compound analysis was performed using MestReNova v7.1.0-9185.

Normal phase thin layer chromatography was conducted on standard commercial aluminium sheets pre-coated with a 0.2 mm layer of silica gel (Merck 60-254), and flash silica column chromatography was performed using a 10 g, 25 g and 50 g pre-packed Biotage Snap columns on a Biotage® Isolera™ Four system.

Microwave-assisted reactions were performed in a Biotage® Initiator 2.5 microwave reactor. ^{1}H NMR spectra were recorded on a Bruker Avance 500 MHz spectrometer using an internal deuterium lock. Chemical shifts were measured in parts per million (ppm) relative to tetramethylsilane (TMS, δ = 0). Data is presented in the following format: chemical shift (multiplicity, coupling constant (*J* in Hz), integration, assignment).
^{13}C NMR spectra were recorded on a Bruker Avance 500 MHz spectrometer using an internal deuterium lock. Chemical shifts were measured in parts per million (ppm) relative to tetramethylsilane (TMS, δ = 0).
LC-MS and HRMS analysis was performed on an Agilent 1200 series HPLC and diode array detector coupled to a 6210 time of flight mass spectrometer with dual multimode APCI/ESI source. Fast4mins: Analytical separation was carried out at 30°C on a Merck Purospher STAR column (RP-18e, 30 x 4 mm) using a flow rate of 1.5 mL/min in a 4-minute gradient elution with detection at 254 nm. The mobile phase was a mixture of methanol (solvent A) and water containing formic acid at 0.1% (solvent B). Gradient elution was as follows: 1:9 (A/B) to 9:1 (A/B) over 2.5 min, 9:1 (A/B) for 1 min, and then reversion back to 1:9 (A/B) over 0.3 min, finally 1:9 (A/B) for 0.2 min. Fast4minsLipo: Analytical separation was carried out at 30°C on a Merck Purospher STAR column (RP-18e, 30 x 4 mm) using a flow rate of 1.5 mL/min in a 4 minute gradient elution with detection at 254 nm. The mobile phase was a mixture of methanol (solvent A) and water containing formic acid at 0.1% (solvent B). Gradient elution was as follows: 1:9 (A/B) to 9:1 (A/B) over 1 min, 9:1 (A/B) for 2.5 min, and then reversion back to 1:9 (A/B) over 0.3 min, finally 1:9 (A/B) for 0.2 min.

The references used for HRMS analysis were: hexakis (2,2-difluroethoxy)phosphazene $[M+H]^+$ 622.02896 and hexakis(1*H*,1*H*,3*H*-tetrafluoropentoxy)phosphazene $[M+H]^+$ 922.009798
All melting points were determined on a Reichert Thermovar melting point apparatus and are uncorrected.

1.3. General procedure for synthesis of small panel of kinase-like scaffolds 4a-4p.

To the appropriate first bromide (1 eq.) and bis(pinocolato)diboron (1.2 eq.) dissolved in 1,4-dioxane (0.5 M) were added KOAc (3 eq.) and tetrakis(triphenylphosphine)palladium(0) (10 mol%) and the reaction mixture was heated under µW irradiation to 120 °C for 45 min. To the crude reaction mixture was added the appropriate second bromide (1 eq.) and a 2M solution Na_2CO_3 (2 eq.) and the reaction mixture was heated under µW irradiation to 120 °C for 30 min. The reaction mixture is then filtered through celite and the organics reduced in vacuo. The residue was then purified by flash silica column chromatography (0-50% EtOAc in cyclohexane) to afford product.

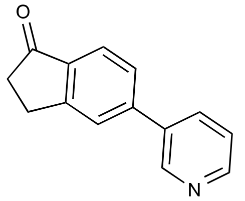

4a

5-(pyridin-3-yl)-2,3-dihydro-1*H*-inden-1-one, 4a. 5-bromo-1-indanone (50 mg, 0.24 mmol) and 3-bromopyridine (20 µL, 0.24 mmol) were reacted respectively, following general procedure. Product obtained as a cream solid (35 mg, 70%). Mp: 96–100 °C; ^1H NMR (500 MHz, Chloroform-d3) δ 8.91 (d, J = 2.3 Hz, 1H), 8.68 (d, J = 2.3 Hz, 1H), 7.94 (d, J = 7.9 Hz, 1H), 7.88 (d, J = 7.9 Hz, 1H), 7.71 – 7.70 (m, 1H), 7.62 – 7.60 (m, 1H), 7.45 – 7.41 (m, 1H), 3.27 – 3.23 (m, 2H), 2.81 – 2.77 (m, 2H); ^{13}C NMR (126 MHz, Chloroform-d3) δ 155.95, 149.44, 148.51, 134.69, 132.06, 128.54, 128.44, 126.73, 125.32, 124.44, 123.68, 123.22, 36.49, 25.90; LC-MS: t_R = 1.39 min; m/z: 210 (M + H)+ ($C_{14}H_{12}NO$); HRMS: (M + H)+ calcd for $C_{14}H_{12}NO$ 210.0919, found 210.0923.

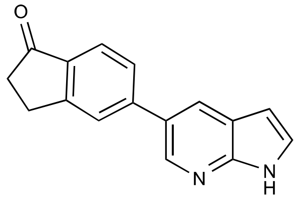

4b

5-(1H-pyrrolo[2,3-b]pyridin-5-yl)-2,3-dihydro-1H-inden-1-on, 4b. 5-bromo-1-indanone (50 mg, 0.24 mmol) and 5-bromo-7-azaindole (47 mg, 0.24 mmol) were reacted respectively, following general procedure. Product obtained as a yellow solid (7 mg, 12%). Mp: 264–266 °C; ^1H NMR (500 MHz, DMSO-d6) δ 11.80 (s, 1H), 8.60 (d, J = 2.2 Hz, 1H), 8.32 (d, J = 2.2 Hz, 1H), 7.94 – 7.92 (m, 1H), 7.78 (d, J = 8.0 Hz, 1H), 7.71 (d, J = 8.0 Hz, 1H), 7.64 – 7.62 (m, 1H), 7.56 – 7.53 (m, 1H), 3.20 – 3.16 (m, 2H), 2.70 – 2.66 (m, 2H); ^{13}C NMR (126 MHz, DMSO-d6) δ

156.64, 148.90, 145.90, 142.25, 135.60, 132.48, 131.97, 129.14, 127.75, 126.73, 125.44, 123.89, 120.17, 100.85, 36.62, 25.99; LC-MS: t_R = 2.63 min; *m/z*: 249 (M + H)$^+$ ($C_{16}H_{13}N_2O$); HRMS: (M + H)$^+$ calcd for $C_{16}H_{13}N_2O$ 249.1028, found 249.1021.

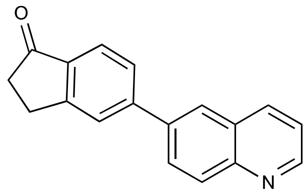

4c

5-(quinolin-6-yl)-2,3-dihydro-1*H*-inden-1-one, 4c. 5-bromo-1-indanone (50 mg, 0.24 mmol) and 6-bromoquinoline (32 μL, 0.24 mmol) were reacted respectively, following general procedure. Product obtained as a white solid (42 mg, 67%). Mp: 147–151 °C; ^{1}H NMR (500 MHz, Chloroform-d3) δ 8.98 (d, J = 4.3 Hz, 1H), 8.27 – 8.25 (m, 1H), 8.23 (d, J = 8.7 Hz, 1H), 8.09 (d, J = 2.1 Hz, 1H), 8.02 (d, J = 8.7 Hz, 1H), 7.90 (d, J = 8.0 Hz, 1H), 7.84 – 7.82 (m, 1H), 7.76 (d, J = 8.0 Hz, 1H), 7.71 – 7.66 (m, 1H), 3.28 – 3.25 (m, 2H), 2.82 – 2.78 (m, 2H); ^{13}C NMR (126 MHz, Chloroform-d3) δ 188.18, 150.95, 148.03, 146.75, 138.34, 136.38, 132.06, 130.26, 129.04, 128.54, 128.39, 127.08, 126.39, 125.58, 124.29, 121.77, 36.56, 25.93; LC-MS: t_R = 2.55 min; *m/z*: 260 (M + H)$^+$ ($C_{18}H_{14}NO$); HRMS: (M + H)$^+$ calcd for $C_{18}H_{14}NO$ 260.1075, found 260.1067.

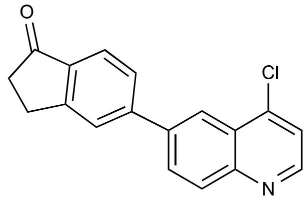

4d

5-(4-chloroquinolin-6-yl)-2,3-dihydro-1*H*-inden-1-one, 4d. 5-bromo-1-indanone (50 mg, 0.24 mmol) and 6-bromo-4-chloroquinoline (58 mg, 0.24 mmol) were reacted respectively, following general procedure. Product obtained as a white solid (70 mg, 100%). Mp: 128–131 °C; ^{1}H NMR (500 MHz, Chloroform-d3) δ 8.84 (d, J = 4.7 Hz, 1H), 8.49 – 8.48 (m, 1H), 8.25 (d, J = 8.7 Hz, 1H), 8.08 (d, J = 8.7 Hz, 1H), 7.92 (d, J = 7.9 Hz, 1H), 7.87 – 7.85 (m, 1H), 7.78 (d, J = 7.9 Hz, 1H), 7.58 (d, J = 4.7 Hz, 1H), 3.30 – 3.27 (m, 2H), 2.82 – 2.79 (m, 2H); ^{13}C NMR (126 MHz, Chloroform-d3) δ 184.38, 150.30, 143.48, 132.14, 131.92, 130.67, 129.99, 128.85, 128.40, 127.21, 125.76, 124.38, 124.03, 122.73, 121.88, 121.38, 36.57, 24.87; LC-MS: t_R = 3.15 min; *m/z*: 294 (M + H)$^+$ ($C_{18}H_{13}ClNO$); HRMS: (M + H)$^+$ calcd for $C_{18}H_{13}ClNO$ 294.0686, found 294.0694.

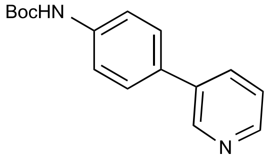

4e

***tert*-butyl (4-(pyridin-3-yl)phenyl)carbamate, 4e.** *Tert*-butyl (4-bromophenyl)carbamate (50 mg, 0.18 mmol) and 3-bromopyridine (18 μL, 0.18 mmol) were reacted respectively, following general procedure. Product obtained as a cream solid (26 mg, 52%). Mp: 147–149 °C; ^{1}H NMR (500 MHz, Chloroform-d3) δ 8.84 – 8.83 (m, 1H), 8.58 – 8.56 (m, 1H), 7.86 (d, J = 7.9 Hz, 1H), 7.54 (d, J =

8.6 Hz, 2H), 7.50 (d, J = 8.6 Hz, 2H), 7.35 (d, J = 7.9 Hz, 1H), 6.65 (s, 1H), 1.55 (s, 9H); ^{13}C NMR (126 MHz, Chloroform-d3) δ 152.63, 148.10, 148.00, 138.53, 136.09, 133.92, 132.36, 127.67, 123.52, 118.96, 80.82, 28.34; LC-MS: t_R = 2.38 min; *m/z*: 271 $(M + H)^+$ ($C_{16}H_{19}N_2O_2$); HRMS: $(M + H)^+$ calcd for $C_{16}H_{19}N_2O_2$ 271.1447, found 271.1441.

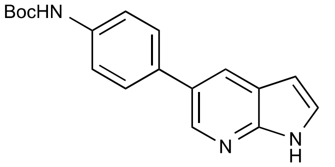

4f

***tert*-butyl (4-(1*H*-pyrrolo[2,3-*b*]pyridin-5-yl)phenyl)carbamate, 4f.** *Tert*-butyl (4-bromophenyl)carbamate (500 mg, 1.57 mmol) and 5-bromo-7-azaindole (309 mg, 1.57 mmol) were reacted respectively, following general procedure. Product obtained as an orange solid (301 mg, 53%). Mp: 187–190 °C; ^{1}H NMR (500 MHz, Chloroform-d3) δ 9.29 (s, 1H), 8.54 (d, J = 2.1 Hz, 1H), 8.10 (d, J = 2.1 Hz, 1H), 7.57 (d, J = 8.6 Hz, 2H), 7.49 (d, J = 8.6 Hz, 2H), 7.38 – 7.36 (m, 1H), 6.59 (s, 1H), 6.58 – 6.55 (m, 1H), 1.56 (s, 9H); ^{13}C NMR (126 MHz, Chloroform-d3) δ 151.82, 142.21, 140.76, 137.49, 134.33, 129.43, 127.88, 127.00, 125.52, 120.18, 119.04, 101.23, 28.37, 24.87; LC-MS: t_R = 2.85 min; *m/z*: 310 $(M + H)^+$ ($C_{18}H_{20}N_3O_2$); HRMS: $(M + H)^+$ calcd for $C_{18}H_{20}N_3O_2$ 310.1556, found 310.1554.

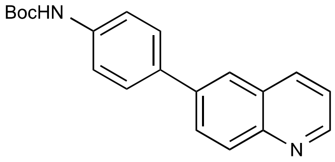

4g

***tert*-butyl (4-(quinolin-6-yl)phenyl)carbamate, 4g.** *Tert*-butyl (4-bromophenyl)carbamate (50 mg, 0.18 mmol) and 6-bromoquinoline (25 µL, 0.18 mmol) were reacted respectively, following general procedure. Product obtained as a cream solid (27 mg, 46%). Mp: 158–160 °C; ^{1}H NMR (500 MHz, Chloroform-d3) δ 8.92 (d, J = 4.2 Hz, 1H), 8.22 (d, J = 8.4 Hz, 1H), 8.19 – 8.15 (m, 1H), 7.99 (d, J = 2.1 Hz, 1H), 7.98 – 7.97 (m, 1H), 7.68 (d, J = 8.5 Hz, 2H), 7.52 (d, J = 8.5 Hz, 2H), 7.44 (d, J = 8.4 Hz, 1H), 6.60 (s, 1H), 1.56 (s, 9H); ^{13}C NMR (126 MHz, Chloroform-d3) δ 150.20, 147.54, 138.72, 136.15, 132.33, 129.86, 128.98, 128.52, 127.96, 126.77, 124.81, 121.46, 118.87, 109.53, 104.08, 28.36; LC-MS: t_R = 2.89 min; *m/z*: 321 $(M + H)^+$ ($C_{20}H_{21}N_2O_2$); HRMS: $(M + H)^+$ calcd for $C_{20}H_{21}N_2O_2$ 321.1603, found 321.1602.

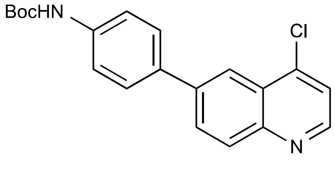

4h

***tert*-butyl (4-(4-chloroquinolin-6-yl)phenyl)carbamate, 4h.** *Tert*-butyl (4-bromophenyl)carbamate (50 mg, 0.18 mmol) and 6-bromo-4-chloroquinoline (44 mg, 0.18 mmol) were reacted respectively, following general procedure. Product obtained as a cream solid (26 mg, 40%). Mp: 124–126 °C; ^{1}H NMR (500 MHz, Chloroform-d3) δ 8.78 (d, J = 4.6 Hz, 1H), 8.39 – 8.38 (m, 1H), 8.19 (d, J =

8.8 Hz, 1H), 8.03 (d, J = 8.8 Hz, 1H), 7.71 (d, J = 8.7 Hz, 2H), 7.55 (d, J = 4.6 Hz, 1H), 7.53 (d, J = 8.7 Hz, 2H), 6.61 (s, 1H), 1.57 (s, 9H); ^{13}C NMR (126 MHz, Chloroform-d3) δ 162.75, 149.52, 148.59, 139.67, 137.83, 137.79, 132.39, 130.29, 129.89, 128.13, 124.56, 121.59, 121.10, 118.90, 99.99, 28.36; LC-MS: t_R = 3.35 min; *m/z*: 355 $(M + H)^+$ ($C_{20}H_{20}ClN_2O_2$); HRMS: $(M + H)^+$ calcd for $C_{20}H_{20}ClN_2O_2$ 355.1213, found 355.1212.

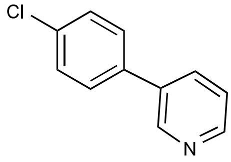

4i

3-(4-chlorophenyl)pyridine, 4i. 1-bromo-4-chlorobenzene (50 mg, 0.26 mmol) and 3-bromopyridine (25 μL, 0.26 mmol) were reacted respectively, following general procedure. Product obtained as a colourless oil (21 mg, 42%). ^{1}H NMR (500 MHz, Chloroform-d3) δ 8.84 – 8.82 (m, 1H), 8.63 – 8.61 (m, 1H), 7.85 (d, J = 7.9 1H), 7.52 (d, J = 8.7 Hz, 2H), 7.46 (d, J = 8.7 Hz, 2H), 7.38 (d, J = 7.9 Hz, 1H); ^{13}C NMR (126 MHz, Chloroform-d3) δ 148.81, 148.14, 136.28, 135.49, 134.38, 134.18, 129.29, 128.39, 123.61; LC-MS: t_R = 2.44 min; *m/z*: 190 $(M + H)^+$ ($C_{11}H_9ClN$); HRMS: $(M + H)^+$ calcd for $C_{11}H_9ClN$ 190.0424, found 190.0418.

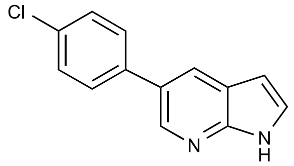

4j

5-(4-chlorophenyl)-1*H*-pyrrolo[2,3-*b*]pyridine, 4j. 1-bromo-4-chlorobenzene (50 mg, 0.26 mmol) and 5-bromo-7-azaindole (52 mg, 0.26 mmol) were reacted respectively, following general procedure. Product obtained as a cream solid (19 mg, 32%). Mp: 210–212 °C; ^{1}H NMR (500 MHz, Chloroform-d3) δ 9.70 (s, 1H), 8.55 (d, J = 2.1 Hz, 1H), 8.12 (d, J = 2.1 Hz, 1H), 7.58 (d, J = 8.6 Hz, 2H), 7.46 (d, J = 8.6 Hz, 2H), 7.42 – 7.40 (m, 1H), 6.60 – 6.58 (m, 1H); ^{13}C NMR (126 MHz, Chloroform-d3) δ 142.16, 138.09, 133.18, 129.07, 128.63, 127.24, 125.82, 120.21, 101.33, 99.99, 29.72; LC-MS: t_R = 3.06 min; *m/z*: 229 $(M + H)^+$ ($C_{13}H_{10}ClN_2$); HRMS: $(M + H)^+$ calcd for $C_{13}H_{10}ClN_2$ 229.0533, found 229.0524.

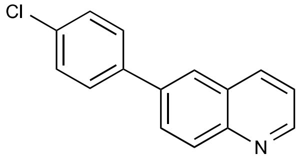

4k

6-(4-chlorophenyl)quinoline, 4k. 1-bromo-4-chlorobenzene (50 mg, 0.26 mmol) and 6-bromoquinoline (36 μL, 0.26 mmol) were reacted respectively, following general procedure. Product obtained as a yellow waxy solid (39 mg, 62%). Mp: 56–60 °C; ^{1}H NMR (500 MHz, Chloroform-d3) δ 8.94 (d, J = 4.2 Hz, 1H), 8.23 – 8.21 (m, 1H), 8.20 (d, J = 8.8 Hz, 1H), 7.98 (d, J = 2.1 Hz, 1H), 7.95 (d, J = 8.8 Hz, 1H), 7.66 (d, J = 8.6 Hz, 2H), 7.48 (d, J = 8.6 Hz, 2H), 7.47 – 7.44 (m, 1H); ^{13}C NMR (126 MHz, Chloroform-d3) δ 150.60, 147.73, 138.76, 138.07, 136.23, 133.95, 130.11, 129.14, 128.87, 128.69, 128.43, 125.46, 121.63; LC-MS: t_R = 3.08

min; *m/z*: 240 $(M + H)^+$ ($C_{15}H_{11}ClN$); HRMS: $(M + H)^+$ calcd for $C_{15}H_{11}ClN$ 240.0580, found 240.0575.

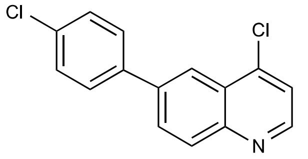

4l

4-chloro-6-(4-chlorophenyl)quinoline, 4l. 1-bromo-4-chlorobenzene (50 mg, 0.26 mmol) and 6-bromo-4-chloroquinoline (63 mg, 0.26 mmol) were reacted respectively, following general procedure. Product obtained as a white solid (35 mg, 49%). Mp: 101–104 °C; ^1H NMR (500 MHz, Chloroform-d3) δ 8.81 (d, J = 4.7 Hz, 1H), 8.40 – 8.39 (m, 1H), 8.21 (d, J = 8.7 Hz, 1H), 8.01 (d, J = 8.7 Hz, 1H), 7.70 (d, J = 8.6 Hz, 2H), 7.55 (d, J = 4.7 Hz, 1H), 7.51 (d, J = 8.6 Hz, 2H); ^{13}C NMR (126 MHz, Chloroform-d3) δ 149.95, 148.56, 142.74, 139.23, 138.47, 135.88, 134.34, 130.56, 129.79, 129.24, 128.83, 126.70, 121.80; LC-MS: t_R = 3.54 min; *m/z*: 274 $(M + H)^+$ ($C_{15}H_{10}Cl_2N$); HRMS: $(M + H)^+$ calcd for $C_{15}H_{10}Cl_2N$ 274.0190, found 274.0185.

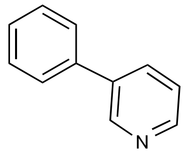

4m

3-phenylpyridine, 4m. Bromobenzene (32 μL, 0.32 mmol) and 3-bromopyridine (31 μL, 0.32 mmol) were reacted respectively, following general procedure. Product obtained as a yellow oil (40 mg, 81%). ^1H NMR (500 MHz, Chloroform-d3) δ 8.88 – 8.86 (m, 1H), 8.61 – 8.59 (m, 1H), 7.89 (d, J = 7.9 Hz, 1H), 7.60 (d, J = 7.4 Hz, 2H), 7.50 (t, J = 7.6 Hz, 2H), 7.44 – 7.40 (m, 1H), 7.38 (d, J = 7.9 Hz, 1H); ^{13}C NMR (126 MHz, Chloroform-d3) δ δ 148.48, 137.84, 136.65, 134.36, 132.14, 129.08, 128.11, 127.16, 123.55; LC-MS: t_R = 1.25 min; *m/z*: 156 $(M + H)^+$ ($C_{11}H_{10}N$); HRMS: $(M + H)^+$ calcd for $C_{11}H_{10}N$ 156.0813, found 156.0816.

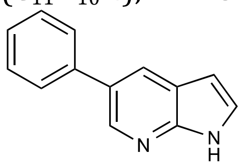

4n

5-phenyl-1*H*-pyrrolo[2,3-*b*]pyridine, 4n. Bromobenzene (32 μL, 0.32 mmol) and 5-bromo-7-azaindole (63 mg, 0.32 mmol) were reacted respectively, following general procedure. Product obtained as a white solid (28 mg, 45%). Mp: 156–160 °C; ^1H NMR (500 MHz, Chloroform-d3) δ 10.07 (s, 1H), 8.60 (d, J = 2.1 Hz, 1H), 8.17 (d, J = 2.1 Hz, 1H), 7.67 (d, J = 7.1 Hz, 2H), 7.50 (t, J = 7.7 Hz, 2H), 7.43 – 7.40 (m, 1H), 7.40 – 7.37 (m, 1H), 6.60 – 6.59 (m, 1H); ^{13}C NMR (126 MHz, Chloroform-d3) δ 148.13, 142.39, 139.63, 132.28, 129.89, 128.92, 127.44, 127.01, 125.65, 120.24, 101.24; LC-MS: t_R = 2.78 min; *m/z*: 195 $(M + H)^+$ ($C_{13}H_{11}N_2$); HRMS: $(M + H)^+$ calcd for $C_{13}H_{11}N_2$ 195.0922, found 195.0917.

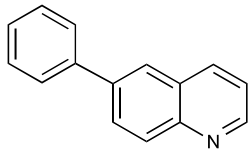

4o

6-phenylquinoline, 4o. Bromobenzene (32 µL, 0.32 mmol) and 6-bromoquinoline (43 µL, 0.32 mmol) were reacted respectively, following general procedure. Product obtained as a yellow solid (58 mg, 87%). Mp: 51–55 °C; ^{1}H NMR (500 MHz, Chloroform-d3) δ 8.94 (d, J = 4.2 Hz, 1H), 8.23 (d, J = 8.4 Hz, 1H), 8.20 (d, J = 8.4 Hz, 1H), 8.03 – 8.01 (m, 1H), 8.00 (d, J = 2.1 Hz, 1H), 7.74 (d, J = 8.4 Hz, 2H), 7.52 (t, J = 7.6 Hz, 2H), 7.47 – 7.45 (m, 1H), 7.44 – 7.41 (m, 1H); ^{13}C NMR (126 MHz, Chloroform-d3) δ 150.38, 147.68, 140.33, 139.34, 136.25, 129.90, 129.24, 128.97, 128.47, 127.76, 127.47, 125.48, 121.48; LC-MS: t_R = 2.80 min; *m/z*: 206 (M + H)$^+$ ($C_{15}H_{12}N$); HRMS: (M + H)$^+$ calcd for $C_{15}H_{12}N$ 206.0970, found 206.0967.

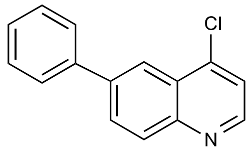

4p

4-chloro-6-phenylquinoline, 4p. Bromobenzene (32 µL, 0.32 mmol) and 6-bromo-4-chloroquinoline (78 mg, 0.32 mmol) were reacted respectively, following general procedure. Product obtained as a white solid (52 mg, 68%). Mp: 79–81 °C; ^{1}H NMR (500 MHz, Chloroform-d3) δ 8.94 (d, J = 4.2 Hz, 1H), 8.23 (d, J = 8.4 Hz, 1H), 8.20 (d, J = 8.4 Hz, 1H), 8.03 – 8.01 (m, 1H), 7.74 (d, J = 8.4 Hz, 2H), 7.54 (d, J = 4.2 Hz, 1H), 7.52 (t, J = 7.6 Hz, 2H), 7.47 – 7.45 (m, 1H); ^{13}C NMR (126 MHz, Chloroform-d3) δ 149.72, 148.50, 142.76, 140.49, 140.03, 130.33, 130.15, 129.05, 128.08, 127.61, 126.70, 121.83, 121.61; LC-MS: t_R = 3.36 min; *m/z*: 240 (M + H)$^+$ ($C_{15}H_{11}ClN$); HRMS: (M + H)$^+$ calcd for $C_{15}H_{11}ClN$ 240.0580, found 240.0575.

1.4. General procedure for synthesis of small panel of kinase-like scaffolds 5a-5d.

To the appropriate first bromide (1 eq.) and bis(pinocolato)diboron (1.2 eq.) dissolved in 1,4-dioxane (0.4 M) were added KOAc (3 eq.) and tetrakis(triphenylphosphine)palladium(0) (10 mol%) and the reaction mixture was heated under µW irradiation to 120 °C for 90 min. To the crude reaction mixture was added the appropriate second bromide (1 eq.) and a 2M solution Na_2CO_3 (2 eq.) and the reaction mixture was heated under µW irradiation to 120 °C for 60 min. The reaction mixture is then filtered through celite and the organics reduced in vacuo. The residue was then purified by flash silica column chromatography (0-50% EtOAc in cyclohexane) to afford product.

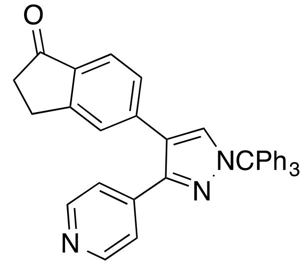

5a

5-(3-(pyridin-4-yl)-1-trityl-1*H*-pyrazol-4-yl)-2,3-dihydro-1*H*-inden-1-one, 5a. 4-(4-bromo-1-trityl-1*H*-pyrazol-3-yl)pyridine (2.00 g, 4.30 mmol) and 5-bromo-1-indanone (0.90 g, 4.30 mmol) were reacted respectively, following general procedure. Product obtained as a white solid (1.27 g, 57%). Mp: 48-51 °C; ^{1}H NMR (500 MHz, MeOD-d4) δ 8.52 (d, J = 6.1 Hz, 2H), 7.71 (d, J = 7.9 Hz, 1H), 7.49 (s, 1H), 7.41 (d, J = 6.1 Hz, 2H), 7.37 (d, J = 7.9 Hz, 1H), 7.38 – 7.36 (m, 10H), 7.26 – 7.25 (m, 1H), 7.25 – 7.24 (m, 5H), 3.12 – 3.08 (m, 2H), 2.74 – 2.70 (m, 2H); ^{13}C NMR (126 MHz, MeOD-d4) δ 206.31, 155.62, 149.81, 145.99, 142.73, 140.95, 139.44, 138.47, 135.81, 133.55, 130.28, 128.23, 128.04, 127.85, 126.57, 123.94, 122.61, 119.66, 36.38, 24.87; LC-MS: t_R = 3.72 min; *m/z*: 518 (M + H)$^+$ ($C_{36}H_{28}N_3O$); HRMS: (M + H)$^+$ calcd for $C_{36}H_{28}N_3O$ 518.2232, found 518.2220.

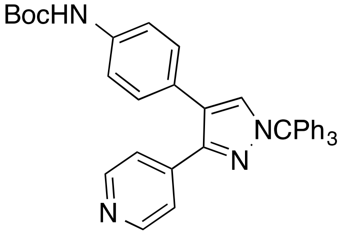

5b

***tert*-butyl (4-(3-(pyridin-4-yl)-1-trityl-1*H*-pyrazol-4-yl)phenyl)carbamate, 5b.** 4-(4-bromo-1-trityl-1*H*-pyrazol-3-yl)pyridine (100 mg, 0.22 mmol) and *tert*-butyl (4-bromophenyl)carbamate (58 mg, 0.22 mmol) were reacted respectively, following general procedure. Product obtained as a white solid (41 mg, 33%). Mp: 204–206 °C; ^{1}H NMR (500 MHz, Chloroform-d3) δ 8.48 (d, J = 6.2 Hz, 2H), 7.44 (d, J = 6.2 Hz, 2H), 7.37 – 7.35 (m, 12H), 7.33 (s, 1H), 7.27 – 7.25 (m, 3H), 7.24 (d, J = 8.5 Hz, 2H), 7.19 (d, J = 8.5 Hz, 2H), 6.53 (s, 1H), 1.54 (s, 9H); ^{13}C NMR (126 MHz, Chloroform-d3) δ 149.70, 147.37, 145.50, 142.98, 141.31, 138.61, 137.48, 134.53, 133.18, 130.32, 129.58, 127.88, 127.75, 127.38, 122.30, 120.05, 118.53, 28.35; LC-MS: t_R = 3.40 min; *m/z*: 579 (M + H)$^+$ ($C_{38}H_{35}N_4O_2$); HRMS: (M + H)$^+$ calcd for $C_{38}H_{35}N_4O_2$ 579.2760, found 579.2932.

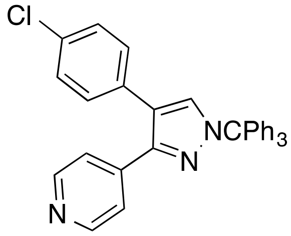

5c

4-(4-(4-chlorophenyl)-1-trityl-1H-pyrazol-3-yl)pyridine, 5c. 4-(4-bromo-1-trityl-1*H*-pyrazol-3-yl)pyridine (100 mg, 0.22 mmol) and 1-bromo-4-chlorobenzene (41 mg, 0.22 mmol) were reacted respectively, following general

procedure. Product obtained as a white solid (52 mg, 49%). Mp: 186–190 °C; ^{1}H NMR (500 MHz, Chloroform-d3) δ 8.51 (d, J = 6.1 Hz, 2H), 7.40 (d, J = 6.1 Hz, 2H), 7.39 (s, 1H), 7.37 – 7.35 (m, 10H), 7.30 (d, J = 8.6 Hz, 2H), 7.25 – 7.23 (m, 5H), 7.20 (d, J = 8.6 Hz, 2H); ^{13}C NMR (126 MHz, Chloroform-d3) δ 149.81, 145.63, 142.85, 141.46, 141.01, 139.90, 138.06, 133.22, 130.29, 130.20, 128.78, 127.96, 127.80, 122.38, 94.63; LC-MS: t_R = 3.67 min; *m/z*: 498 $(M + H)^+$ ($C_{33}H_{25}ClN_3$); HRMS: $(M + H)^+$ calcd for $C_{33}H_{25}ClN_3$ 498.1737, found 498.1732.

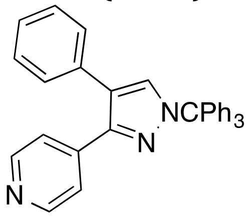

5d

4-(4-phenyl-1-trityl-1*H*-pyrazol-3-yl)pyridine, 5d. 4-(4-bromo-1-trityl-1*H*-pyrazol-3-yl)pyridine (100 mg, 0.22 mmol) and bromobenzene (21 mL, 0.22 mmol) were reacted respectively, following general procedure. Product obtained as a white solid (65 mg, 65%). Mp: 238–242 °C; ^{1}H NMR (500 MHz, Chloroform-d3) δ 8.48 (d, J = 6.2 Hz, 2H), 7.43 (d, J = 6.2 Hz, 2H), 7.40 (s, 1H), 7.37 – 7.34 (m, 12H), 7.35 (d, J = 7.8 Hz, 2H), 7.33 – 7.30 (m, 1H), 7.27 – 7.24 (m, 3H), 7.25 (t, J = 7.6 Hz, 2H); ^{13}C NMR (126 MHz, Chloroform-d3) δ 149.70, 148.23, 142.96, 142.34, 141.20, 140.70, 138.47, 133.34, 130.32, 128.97, 128.56, 127.90, 127.76, 122.38, 99.99; LC-MS: t_R = 3.67 min; *m/z*: 464 $(M + H)^+$ ($C_{33}H_{26}N_3$); HRMS: $(M + H)^+$ calcd for $C_{33}H_{26}N_3$ 464.2127, found 464.2119.

2. NMR Spectra

2.1. ^{1}H NMR

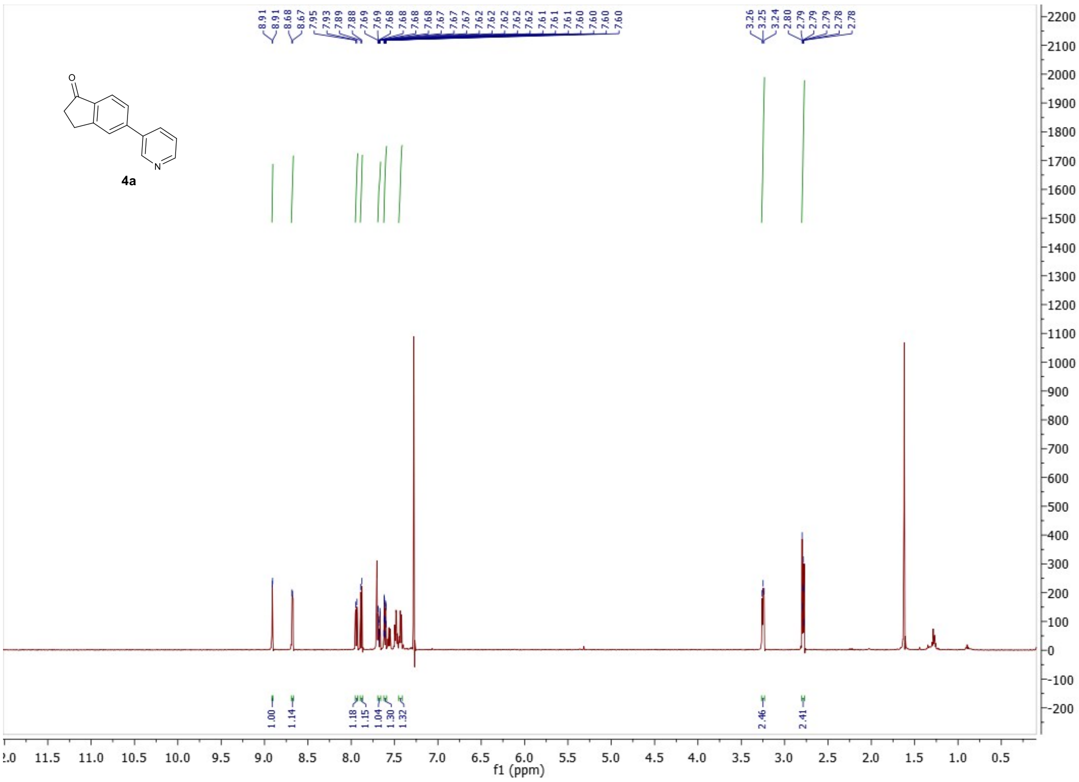

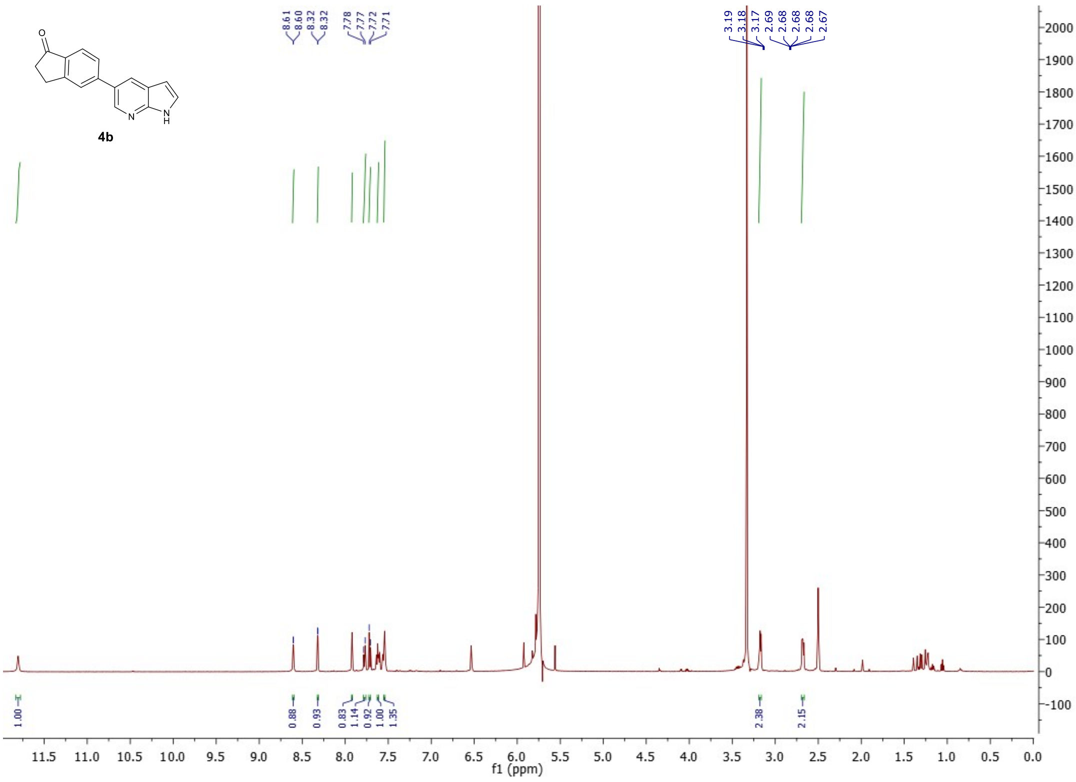

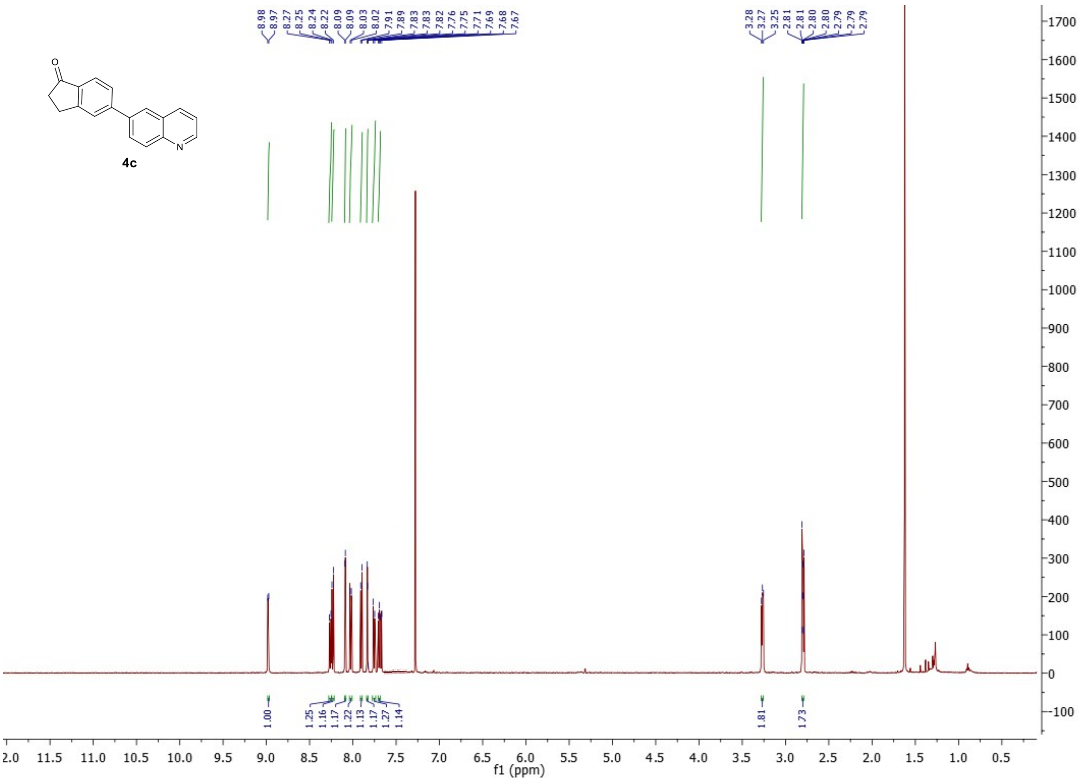
8.98
8.97
8.27
8.25
8.24
8.22
8.09
8.09
8.03
8.02
7.91
7.89
7.83
7.83
7.82
7.76
7.75
7.71
7.69
7.68
7.67
3.28
3.27
3.25
2.81
2.80
2.80
2.79
2.79
2.79
O
N
1.00
1.25
1.16
1.17
1.22
1.13
1.17
1.27
1.14
1.81
1.73
f1 (ppm)

4c

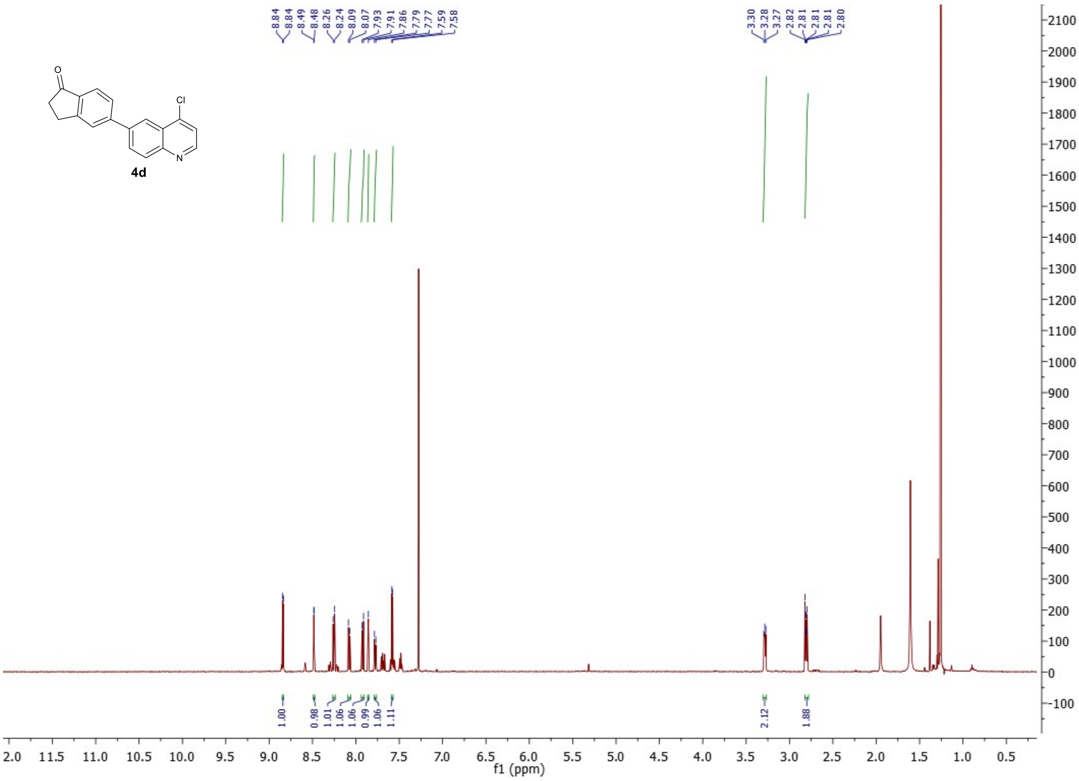
8.84
8.84
8.49
8.48
8.26
8.24
8.09
8.07
7.93
7.91
7.86
7.79
7.77
7.59
7.58
3.30
3.28
3.27
2.82
2.81
2.81
2.80
O
Cl
N
1.00
0.98
1.01
1.06
1.06
0.99
1.06
1.11
2.12
1.88
f1 (ppm)

4d

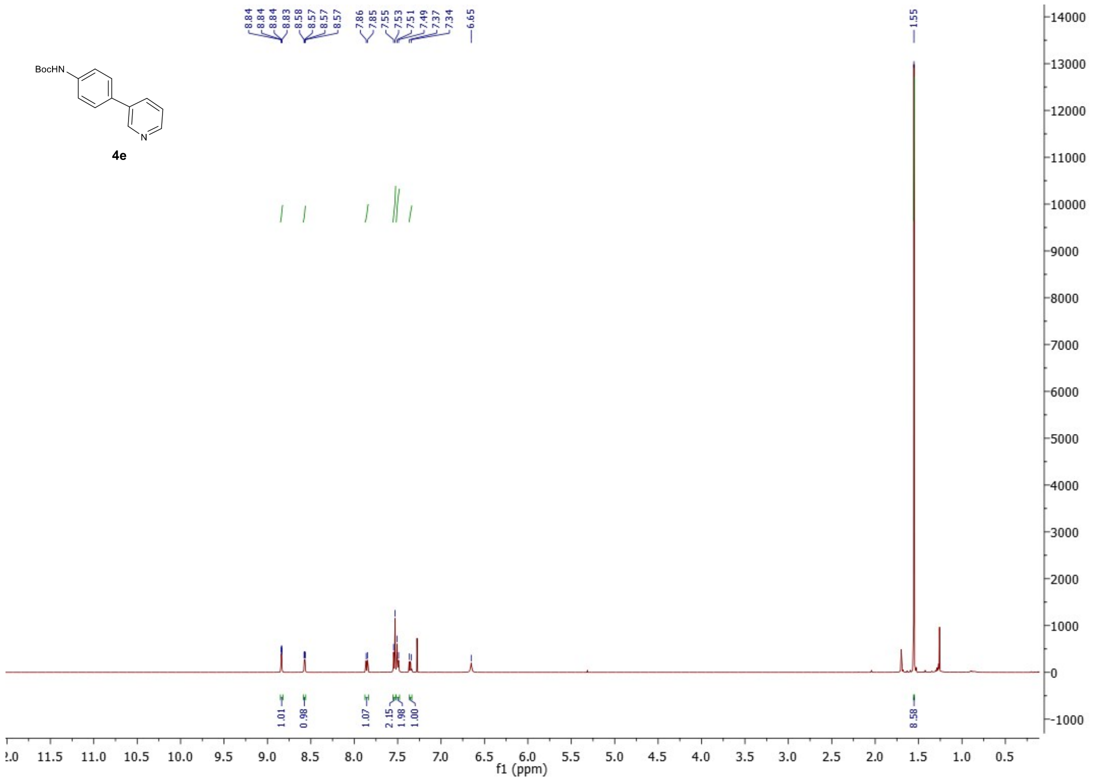
BocHN
N
4e
f1 (ppm)

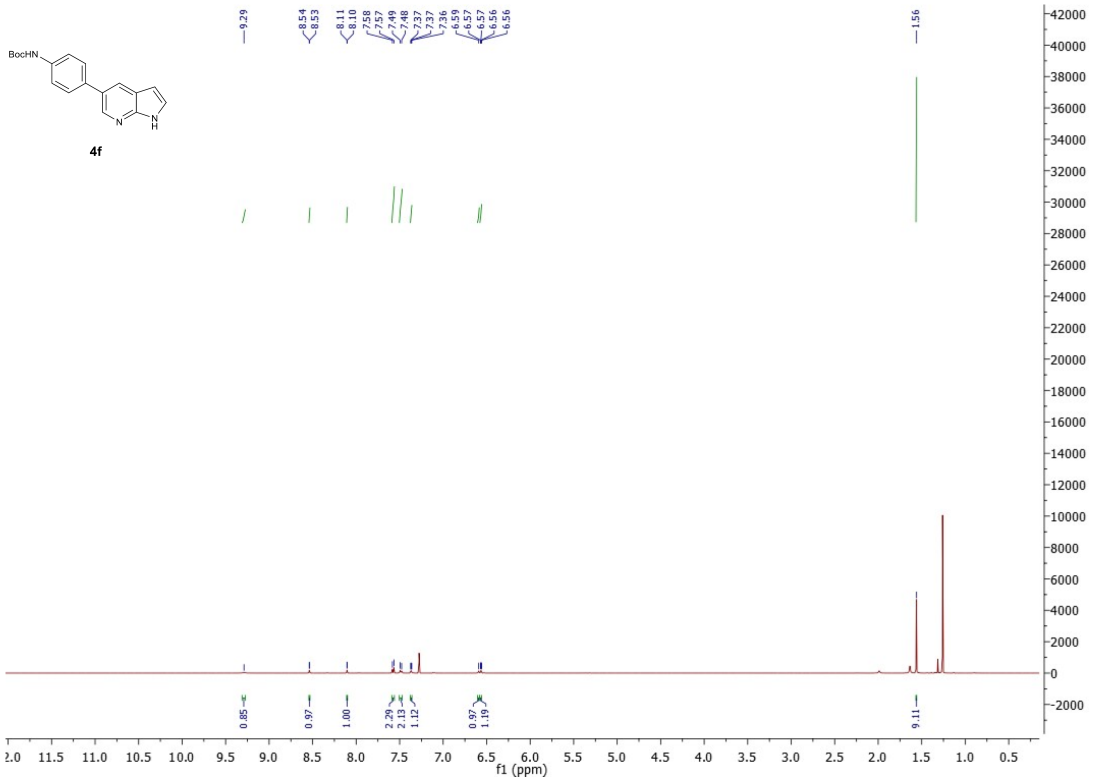
BocHN
N
H
4f
f1 (ppm)

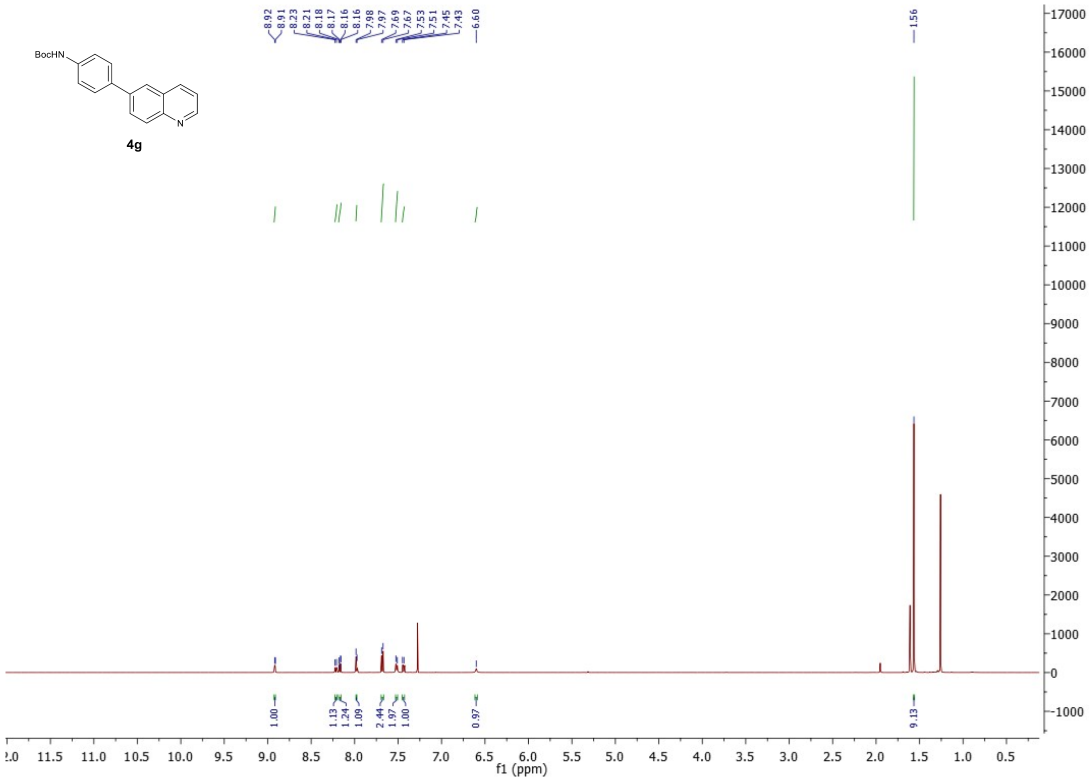
BocHN
N
4g
8.92 8.91 8.23 8.21 8.18 8.17 8.16 8.16 7.98 7.97 7.69 7.67 7.53 7.51 7.45 7.43
6.60
1.56
1.00 1.13 1.24 1.09 2.44 1.97 1.00 0.97 9.13
f1 (ppm)

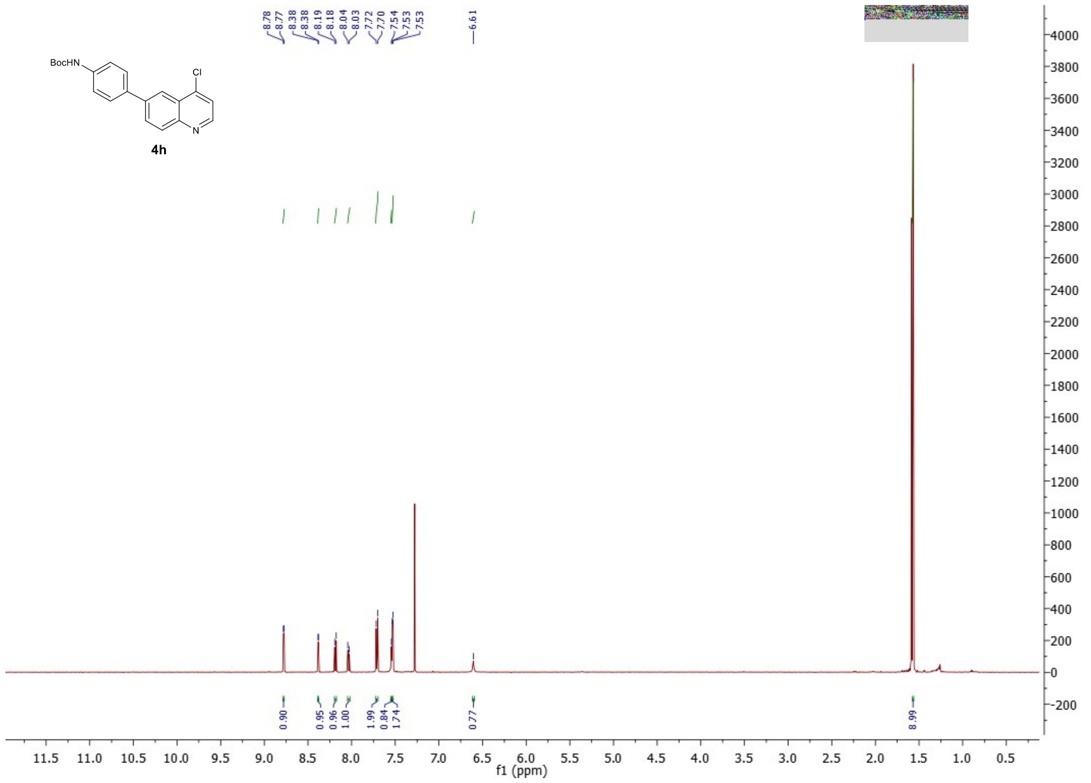
BocHN
Cl
N
4h
8.78 8.77 8.38 8.38 8.19 8.18 8.04 8.03 7.72 7.70 7.54 7.53 7.53
6.61
0.90 0.95 0.96 1.00 1.99 0.84 1.74 0.77 8.99
f1 (ppm)

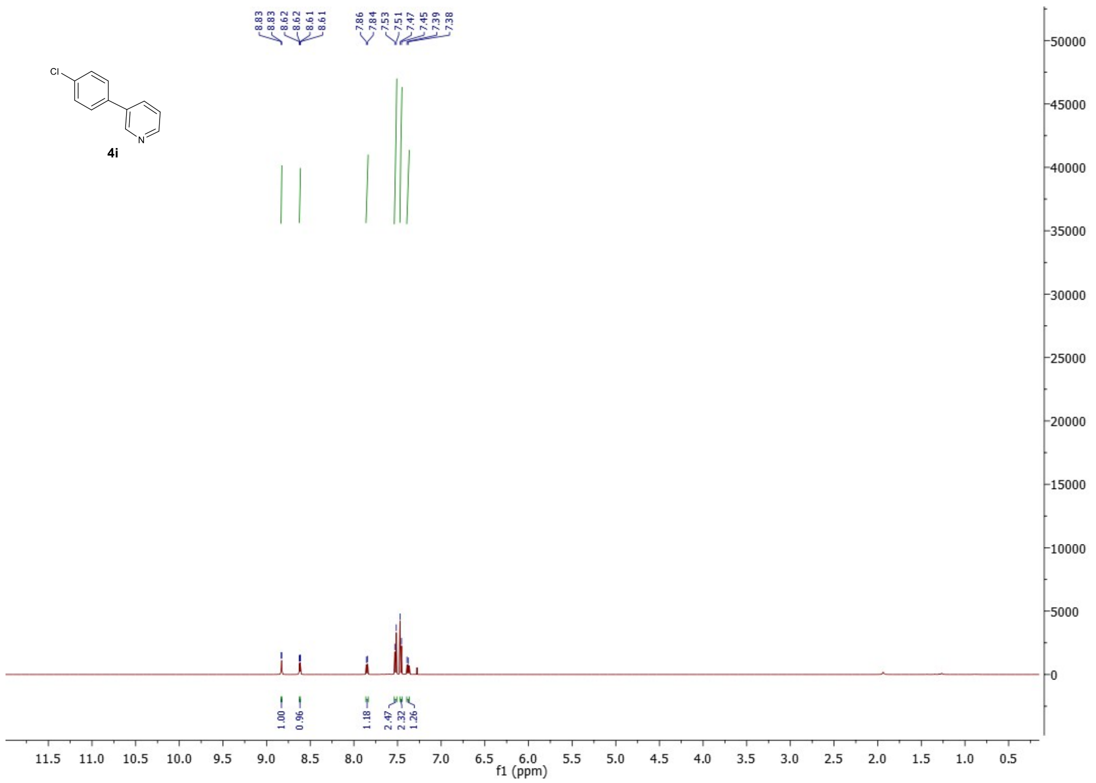
4i
Cl
N
f1 (ppm)

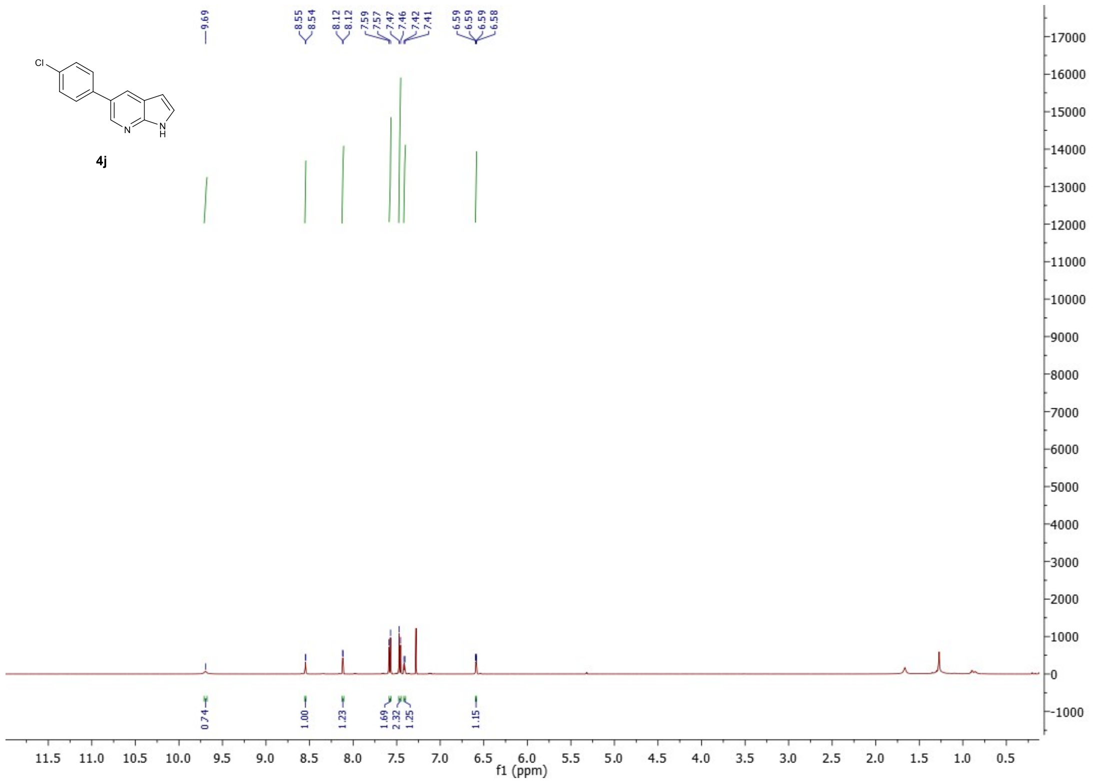
4j
Cl
N
H
f1 (ppm)

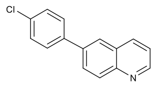
Cl
N

4k

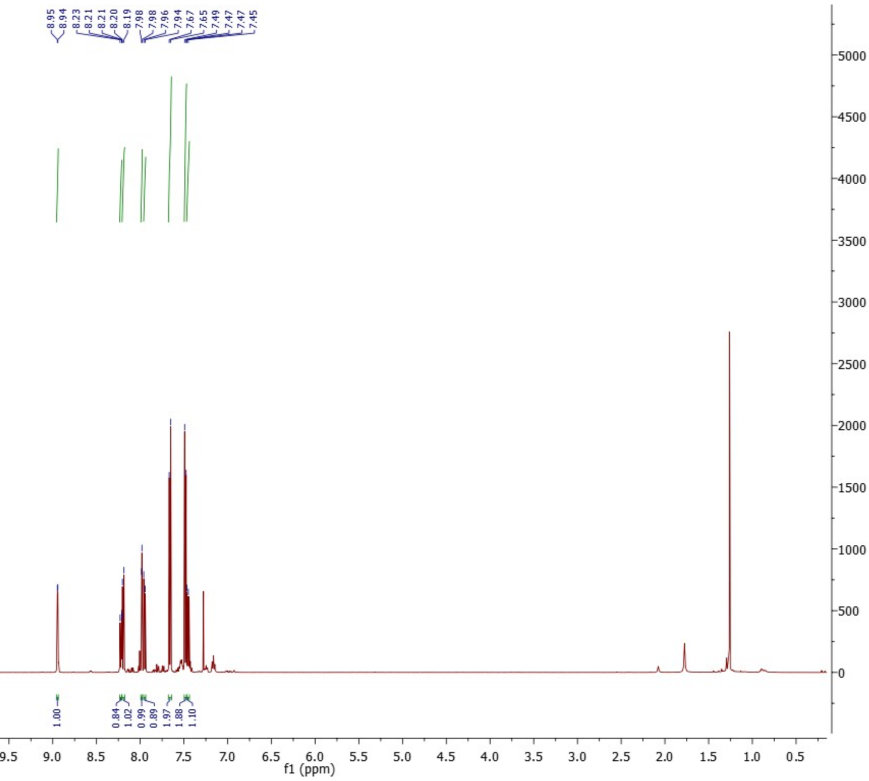
8.95 8.94 8.23 8.21 8.21 8.20 8.19 7.98 7.96 7.94 7.67 7.65 7.49 7.47 7.47 7.45
1.00 0.84 1.02 0.99 0.89 1.97 1.88 1.10
f1 (ppm)

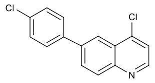
Cl
Cl
N

4l

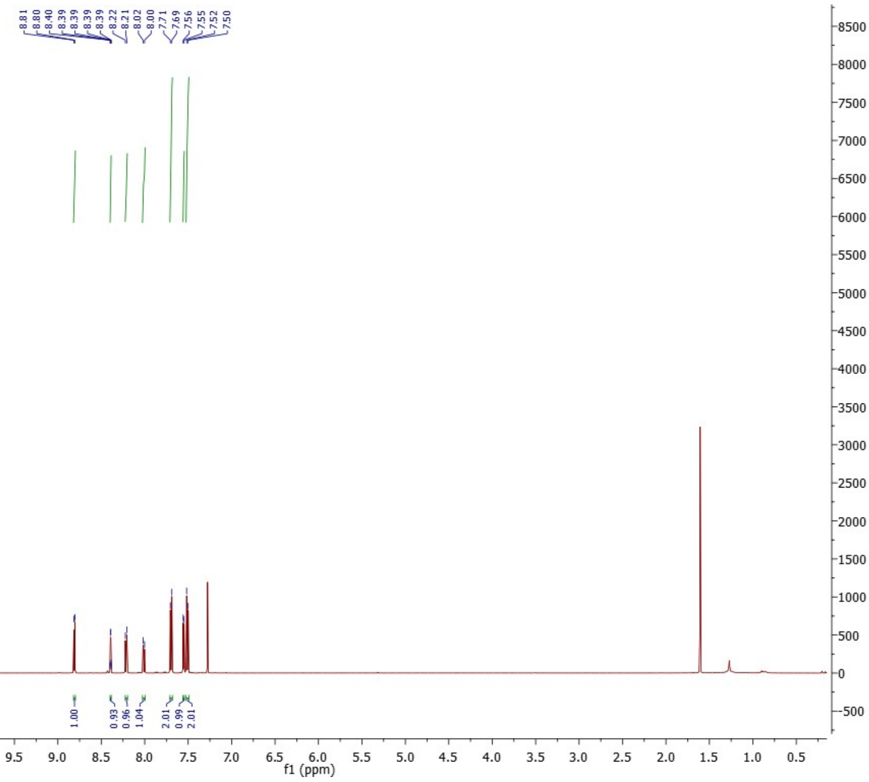
8.81 8.80 8.40 8.39 8.39 8.39 8.22 8.21 8.02 8.00 7.71 7.69 7.56 7.55 7.52 7.50
1.00 0.93 0.96 1.04 2.01 0.99 2.01
f1 (ppm)

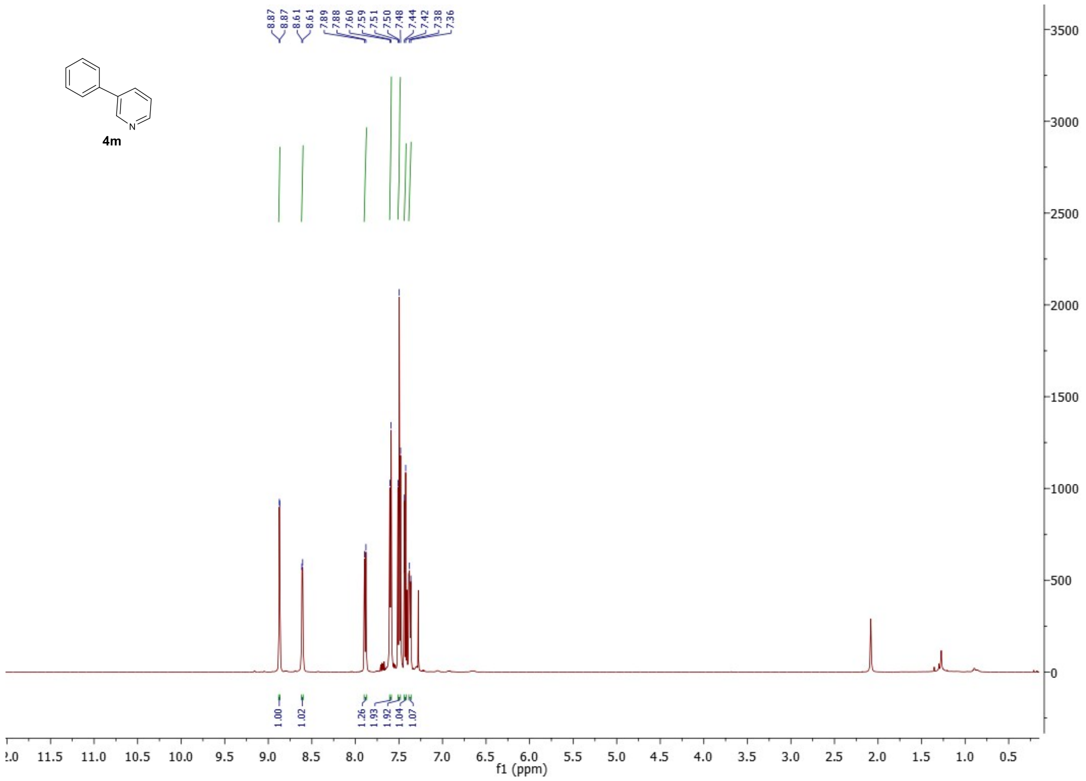
4m
f1 (ppm)

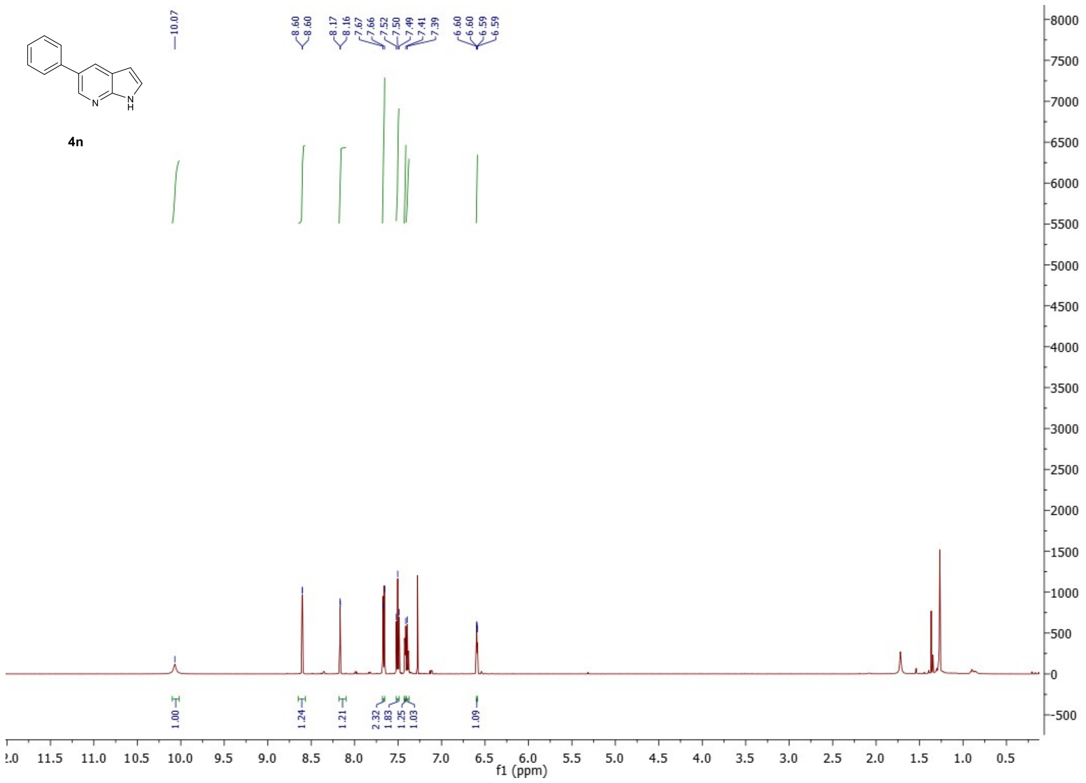
4n
f1 (ppm)

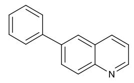

4o

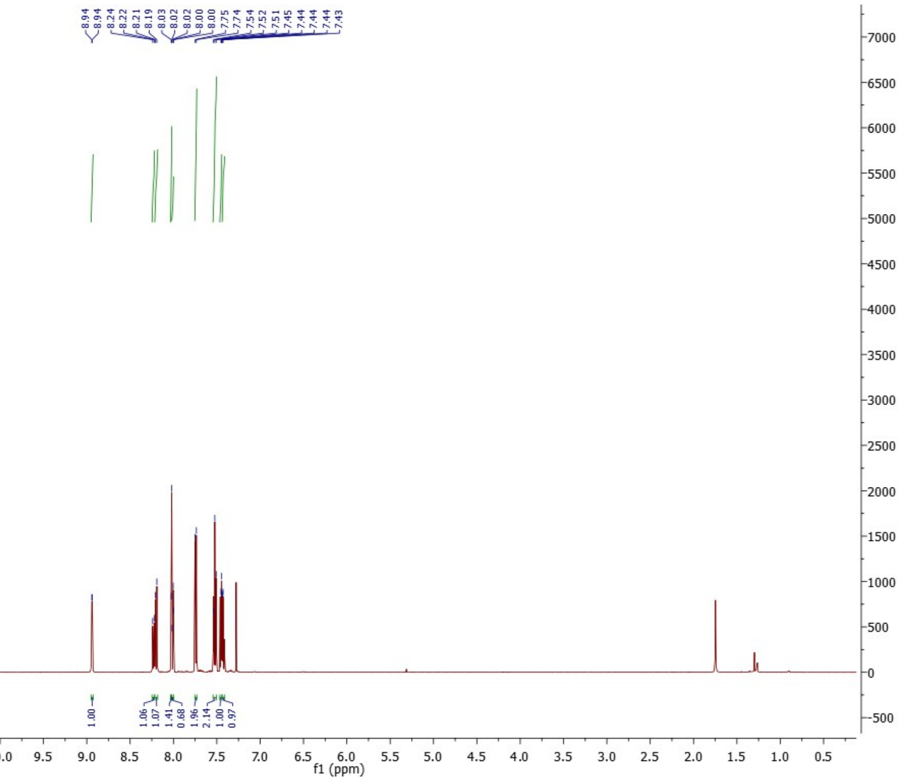
8.94
8.94
8.24
8.22
8.21
8.19
8.03
8.02
8.02
8.00
8.00
7.75
7.74
7.54
7.52
7.51
7.45
7.44
7.44
7.43
1.00
1.06
1.07
1.41
0.68
1.96
2.14
1.00
0.97
f1 (ppm)

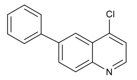

4p

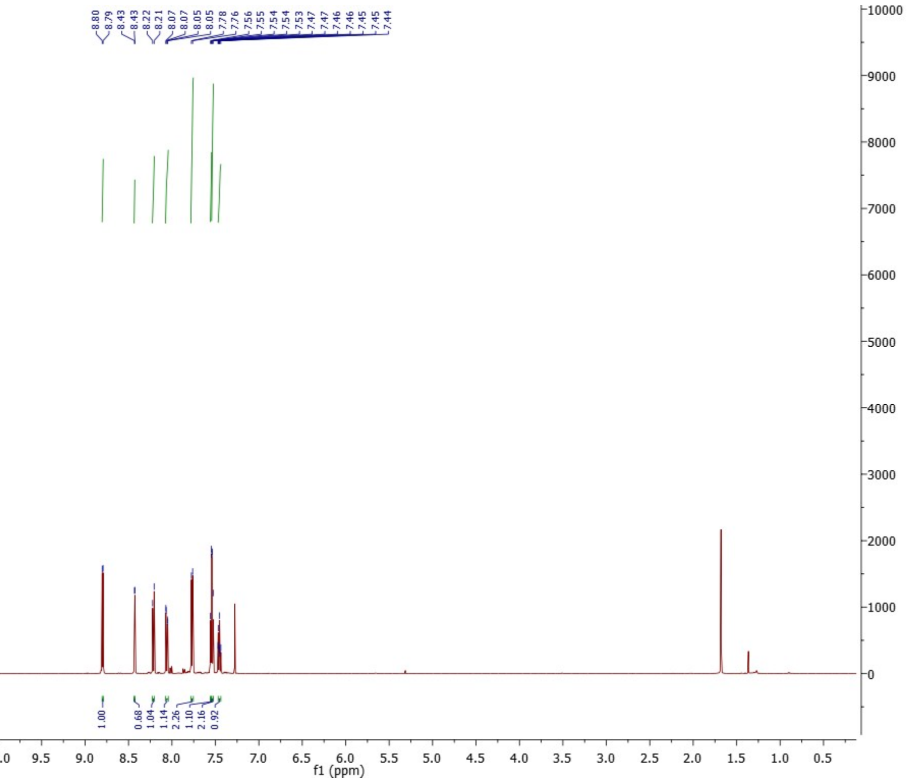
8.80
8.79
8.43
8.22
8.21
8.07
8.07
8.05
8.05
7.78
7.76
7.56
7.55
7.54
7.54
7.53
7.47
7.46
7.46
7.45
7.44
1.00
0.68
1.04
1.14
2.26
1.10
2.16
0.92
f1 (ppm)

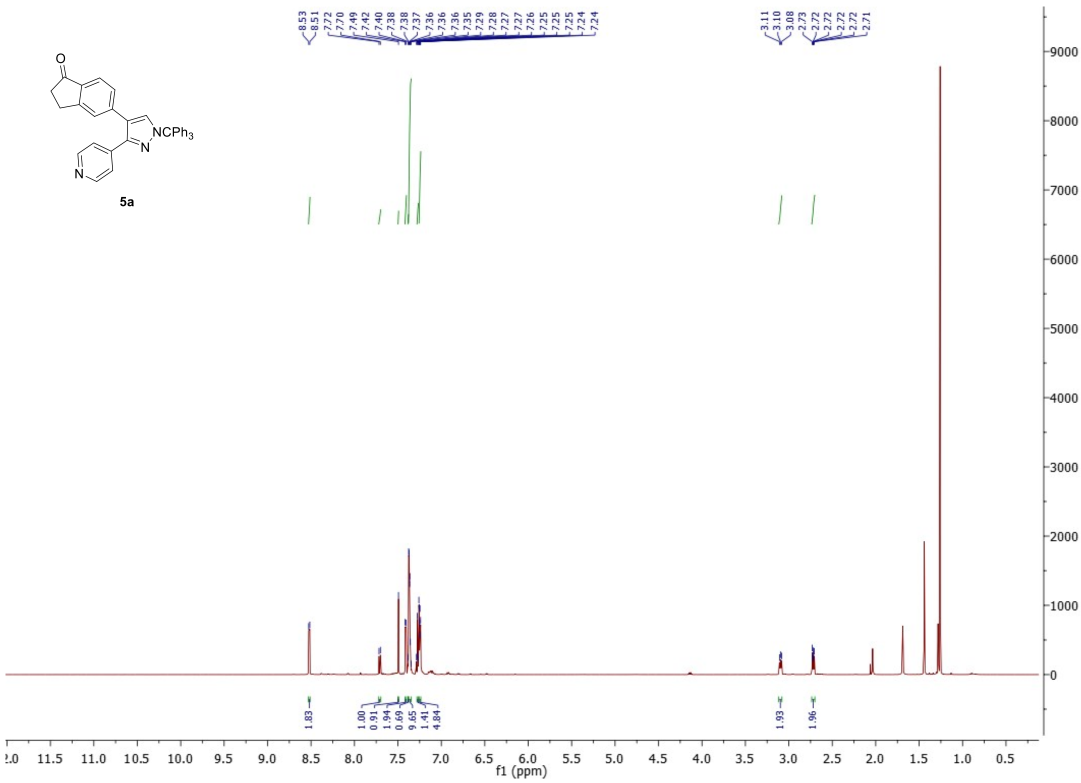
O
NCPh3
N
N
5a
f1 (ppm)

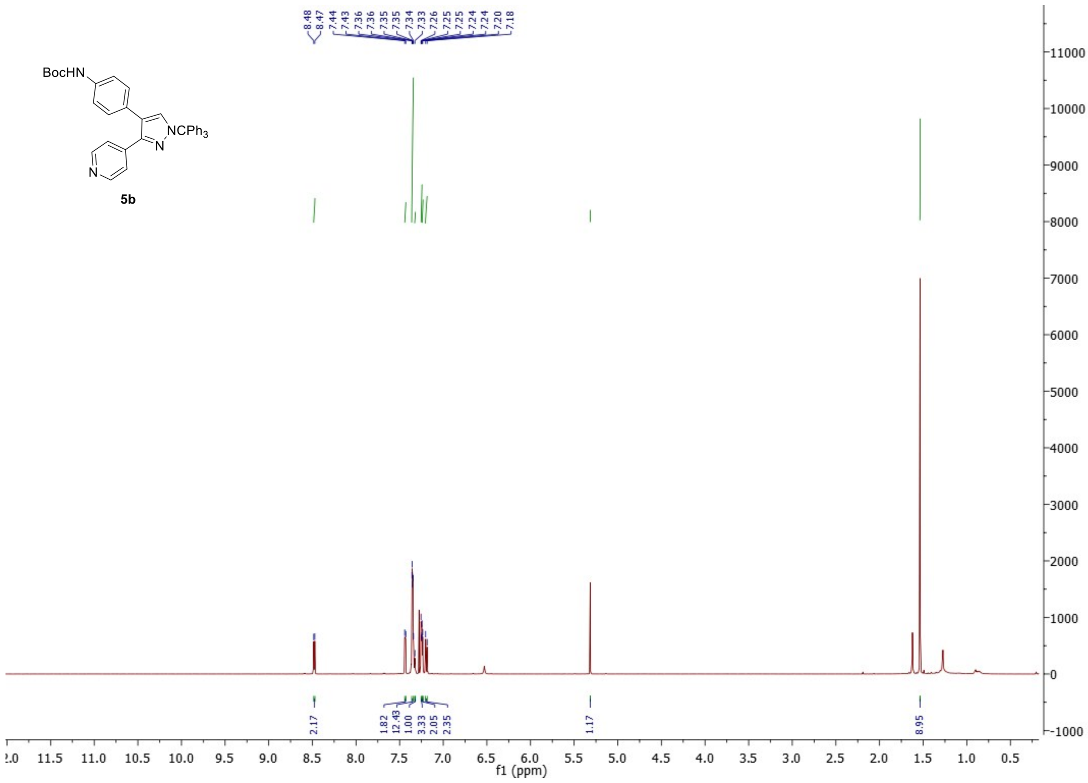
BocHN
NCPh3
N
N
5b
f1 (ppm)

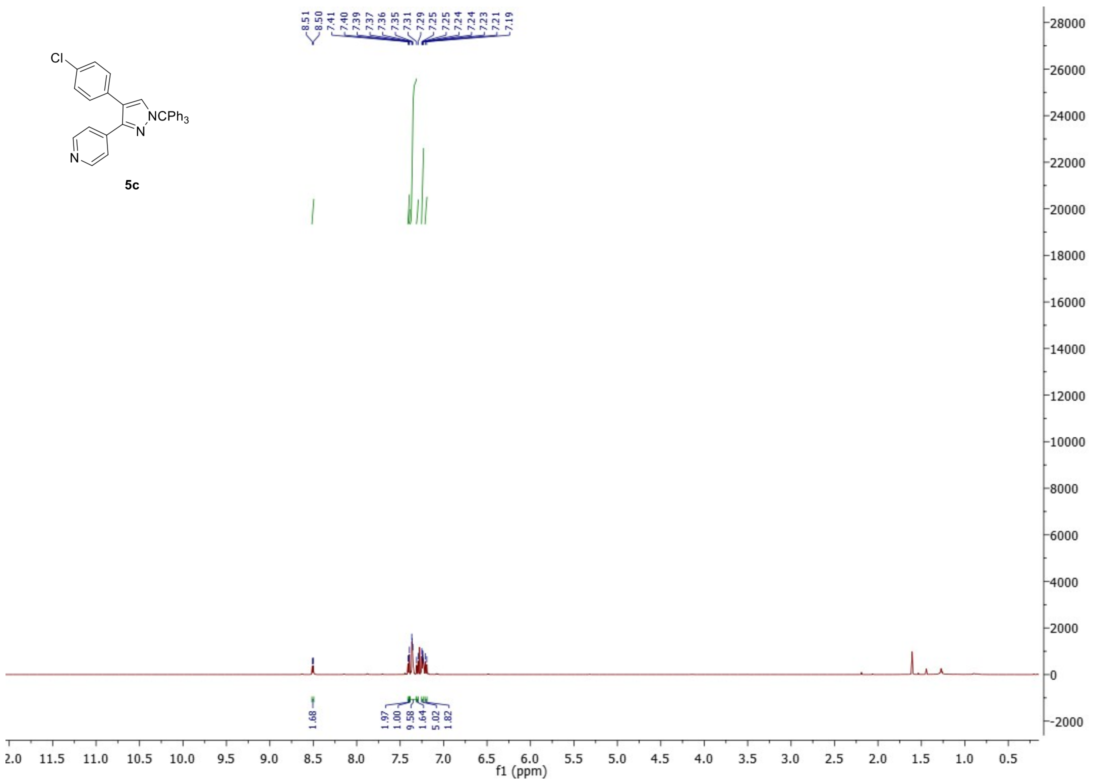

5c

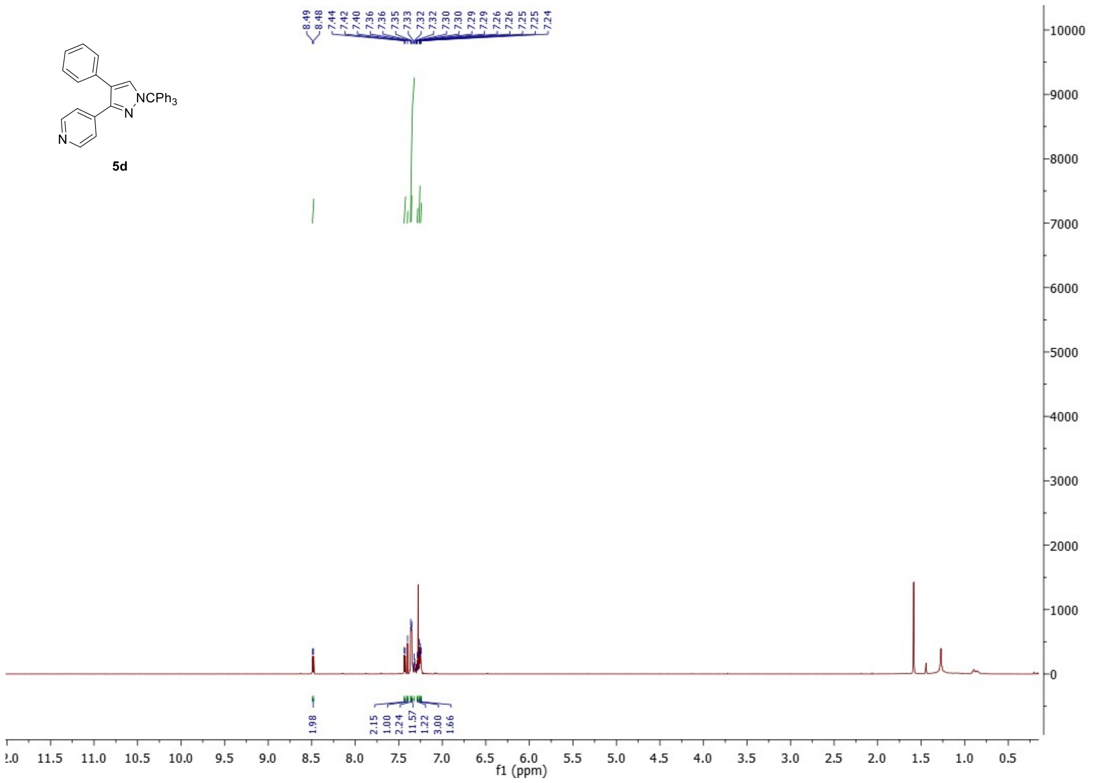

5d

2.2. ^{13}C NMR

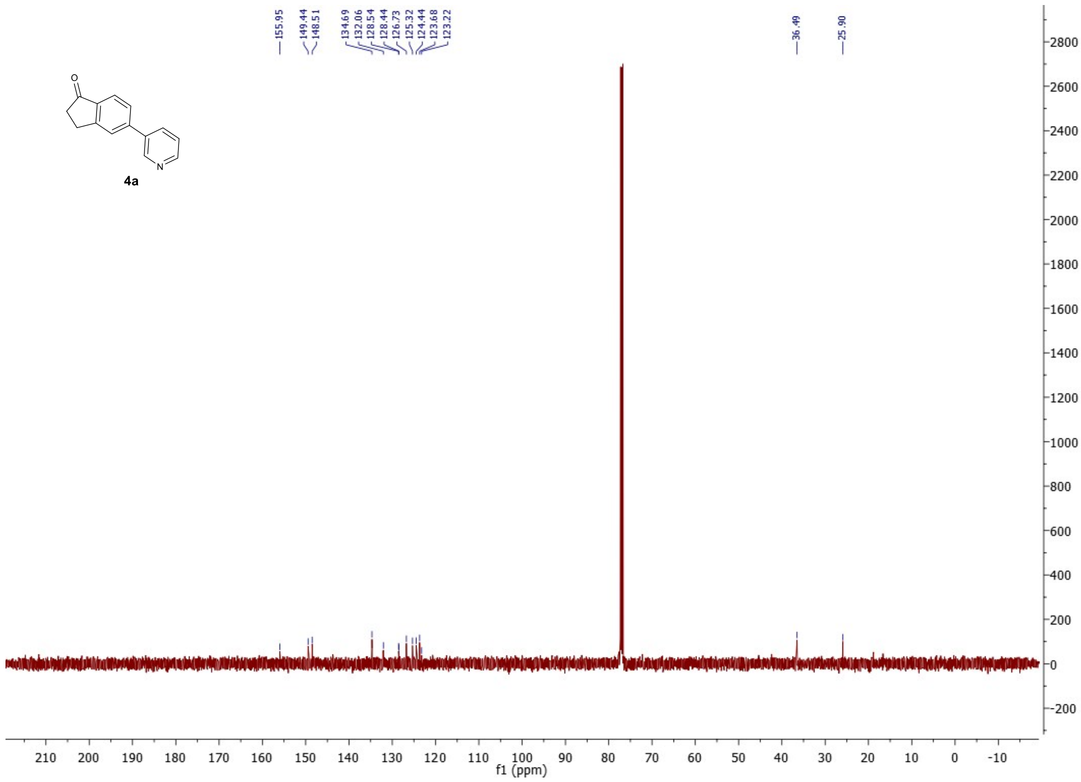
155.95
149.44
148.51
134.69
132.06
128.54
128.44
126.73
125.32
124.44
123.68
123.22
36.49
25.90
O
N
4a
f1 (ppm)

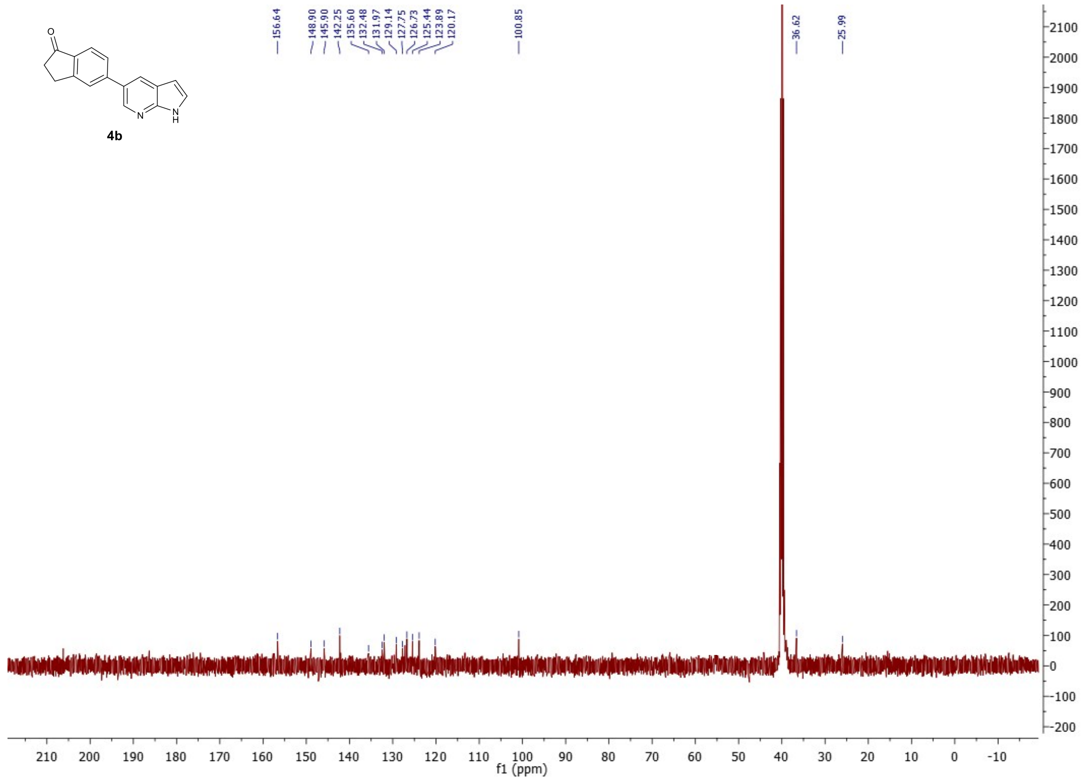
156.64
148.90
145.90
142.25
135.60
132.48
131.97
129.14
127.75
126.73
125.44
123.89
120.17
100.85
36.62
25.99
O
N
H
4b
f1 (ppm)

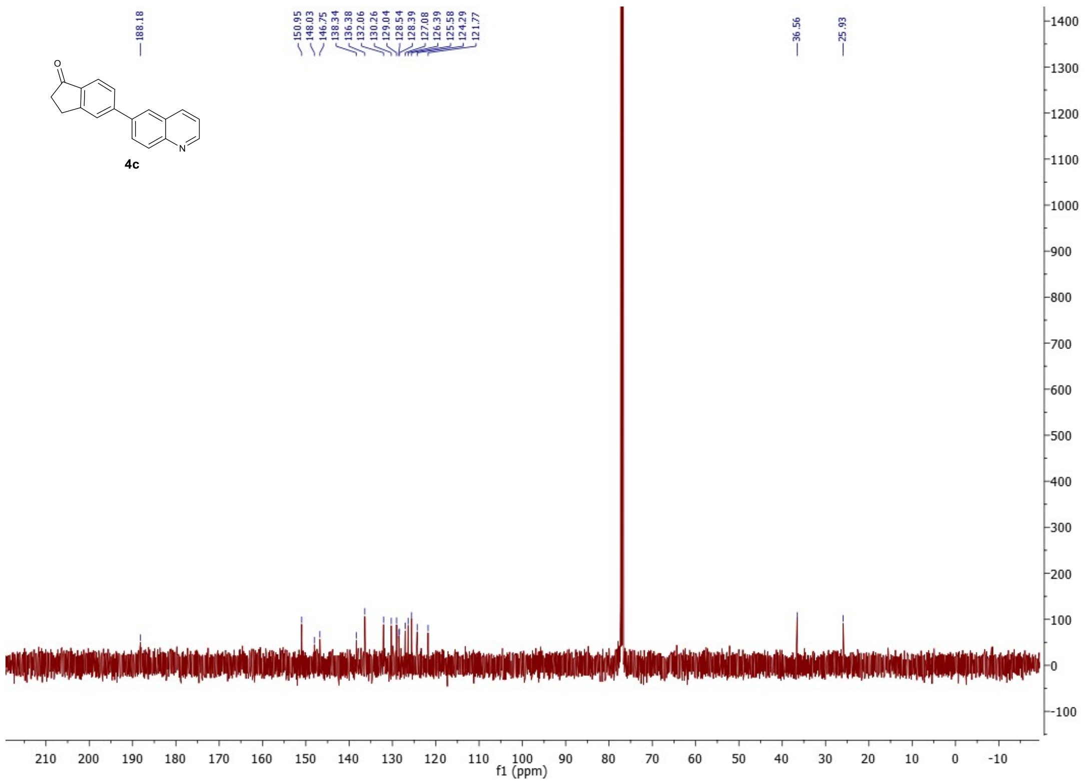
188.18
150.95
148.03
146.75
138.34
136.38
132.06
130.26
129.04
128.54
128.39
127.08
126.39
125.58
124.29
121.77
36.56
25.93
4c
f1 (ppm)

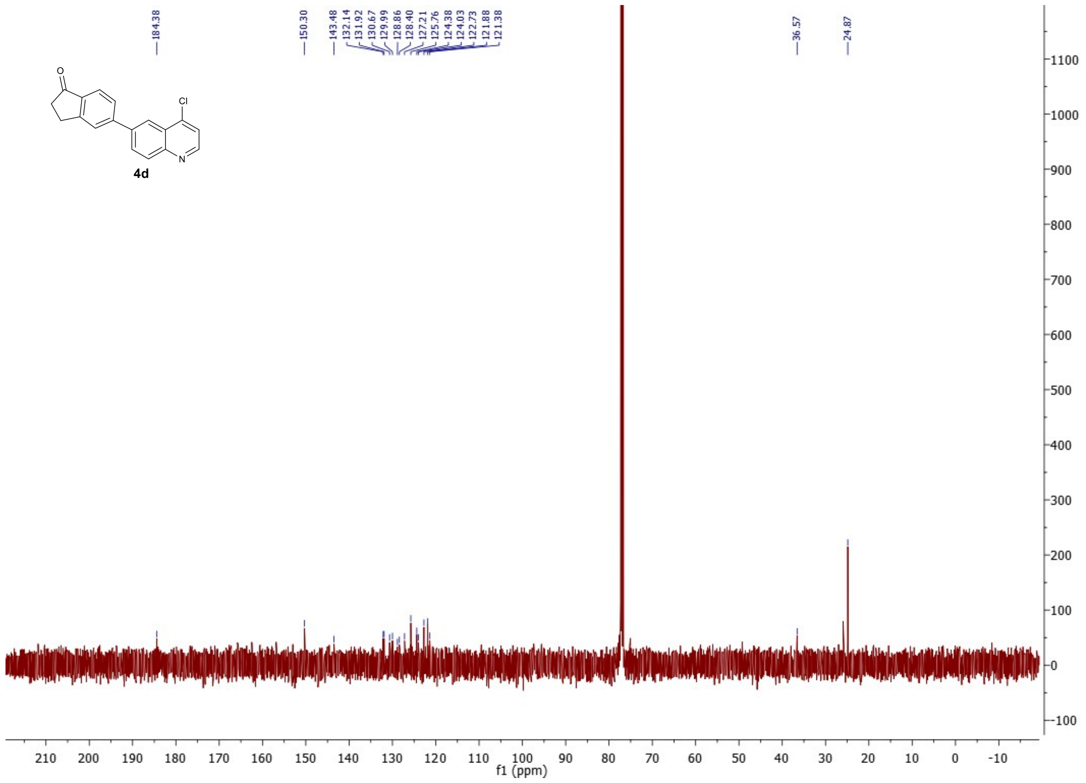
184.38
150.30
143.48
132.14
131.92
130.67
129.99
128.86
128.40
127.21
125.76
124.38
124.03
122.73
121.88
121.38
36.57
24.87
Cl
4d
f1 (ppm)

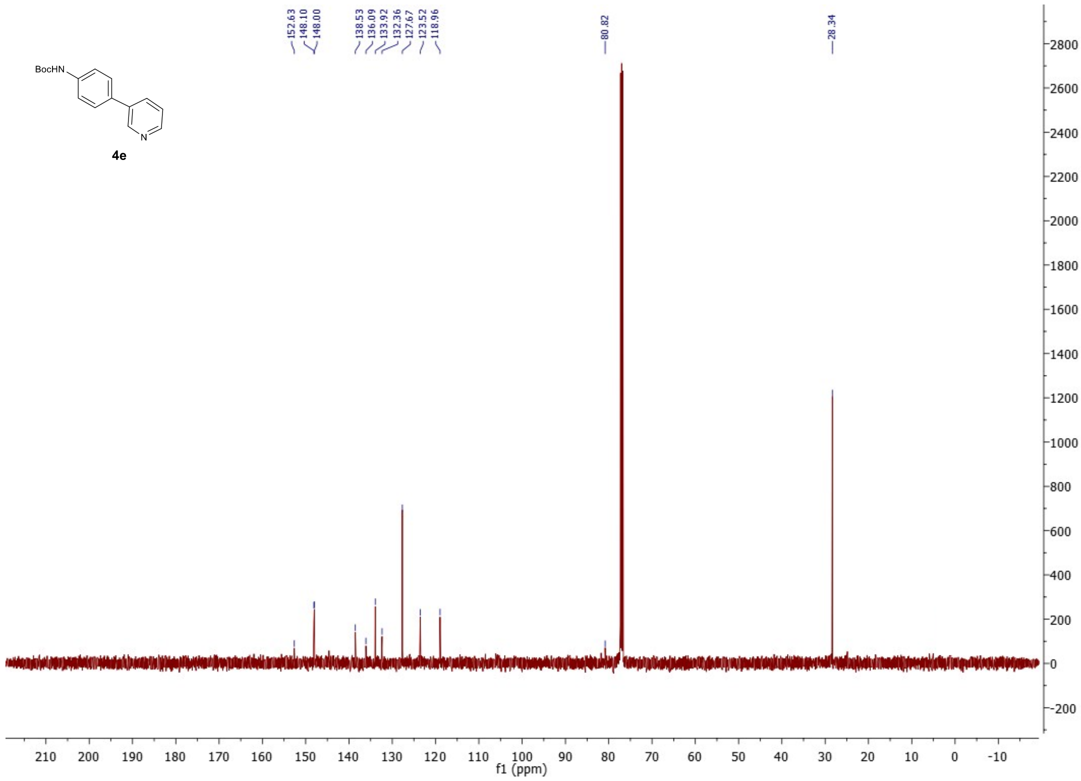
152.63
148.10
148.00
138.53
136.09
133.92
132.36
127.67
123.52
118.96
80.82
28.34
BocHN
N
4e
2800
2600
2400
2200
2000
1800
1600
1400
1200
1000
800
600
400
200
0
-200
210 200 190 180 170 160 150 140 130 120 110 100 90 80 70 60 50 40 30 20 10 0 -10
f1 (ppm)

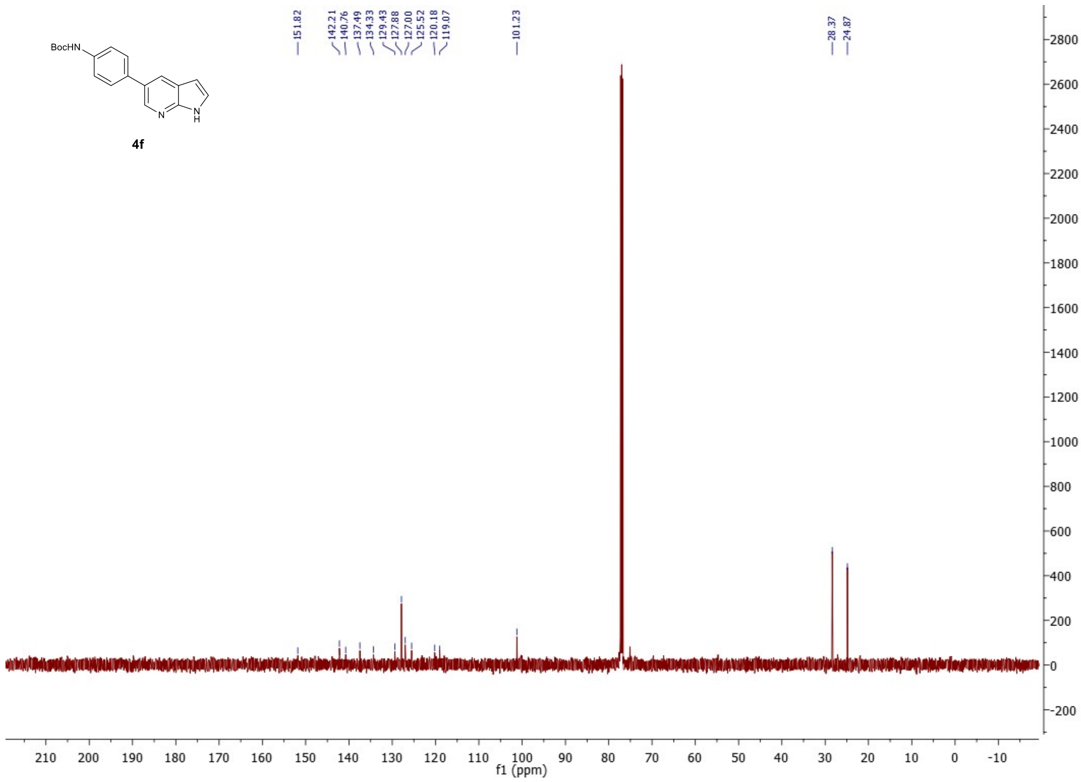
151.82
142.21
140.76
137.49
134.33
129.43
127.88
127.00
125.52
120.18
119.07
101.23
28.37
24.87
BocHN
N
H
4f
2800
2600
2400
2200
2000
1800
1600
1400
1200
1000
800
600
400
200
0
-200
210 200 190 180 170 160 150 140 130 120 110 100 90 80 70 60 50 40 30 20 10 0 -10
f1 (ppm)

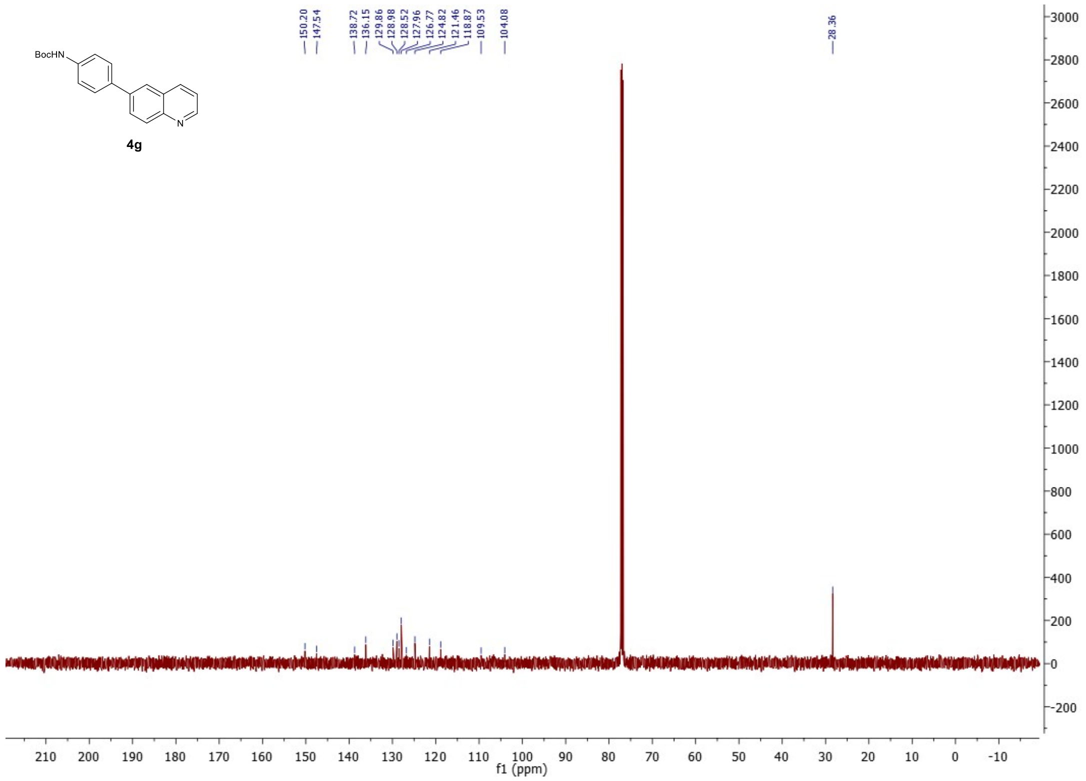
150.20
147.54
138.72
136.15
129.86
128.98
128.52
127.96
126.77
124.82
121.46
118.87
109.53
104.08
28.36
BocHN
N
4g
f1 (ppm)

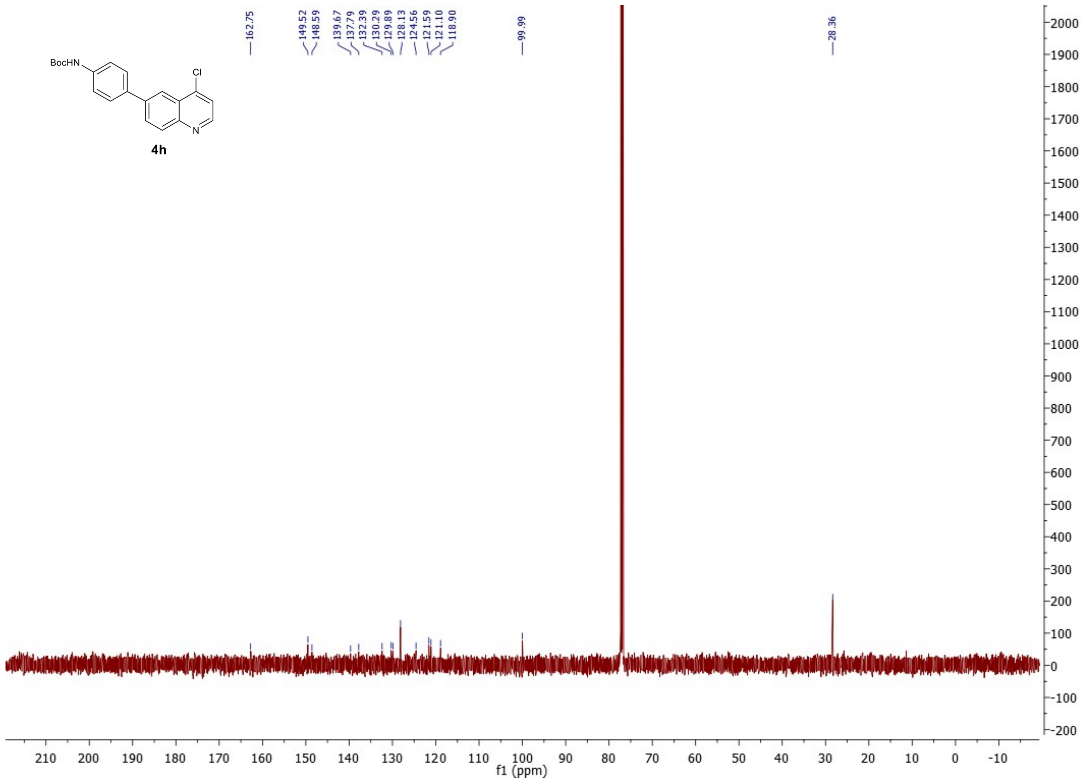
162.75
149.52
148.59
139.67
137.79
132.39
130.29
129.89
128.13
124.56
121.59
121.10
118.90
99.99
28.36
BocHN
Cl
N
4h
f1 (ppm)

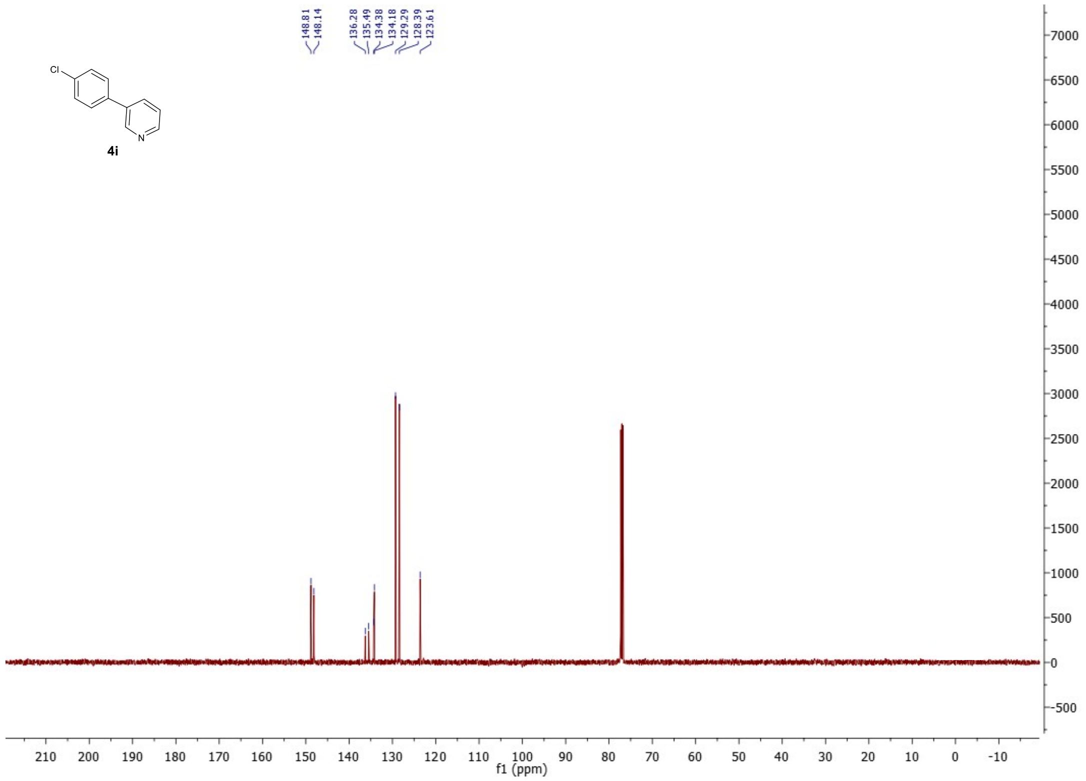
148.81
148.14
136.28
135.49
134.38
134.18
129.29
128.39
123.61
Cl
N
4i
f1 (ppm)

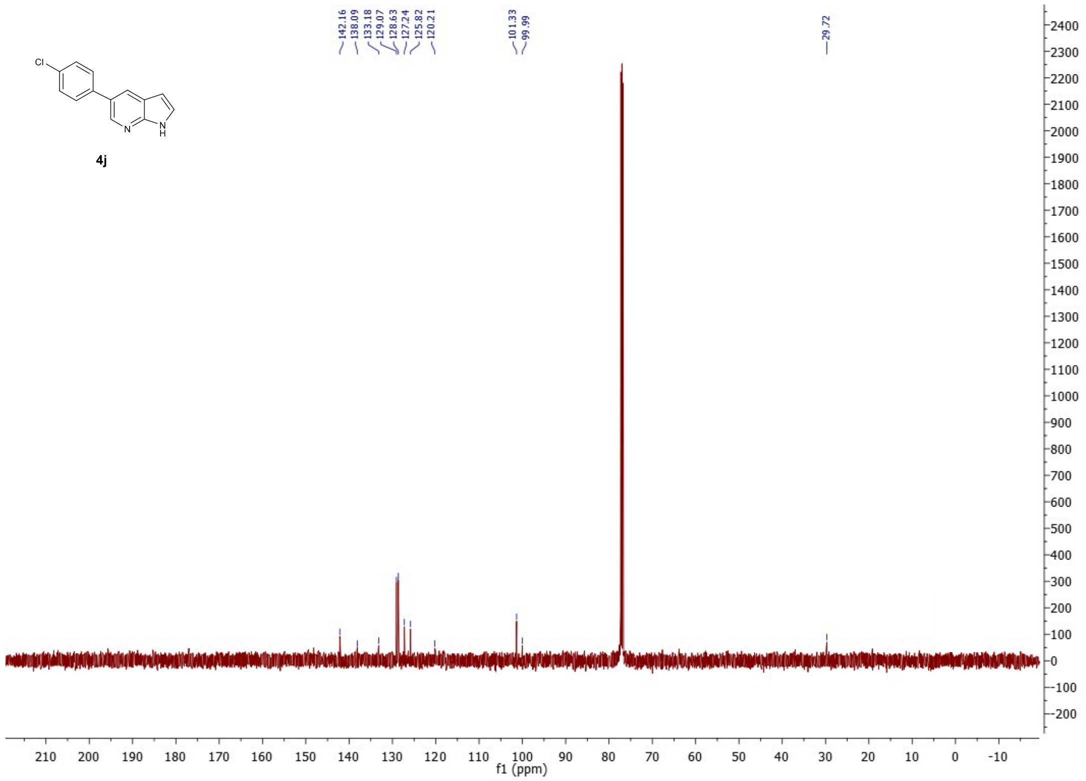
142.16
138.09
133.18
129.07
128.63
127.24
125.82
120.21
101.33
99.99
29.72
Cl
N
H
4j
f1 (ppm)

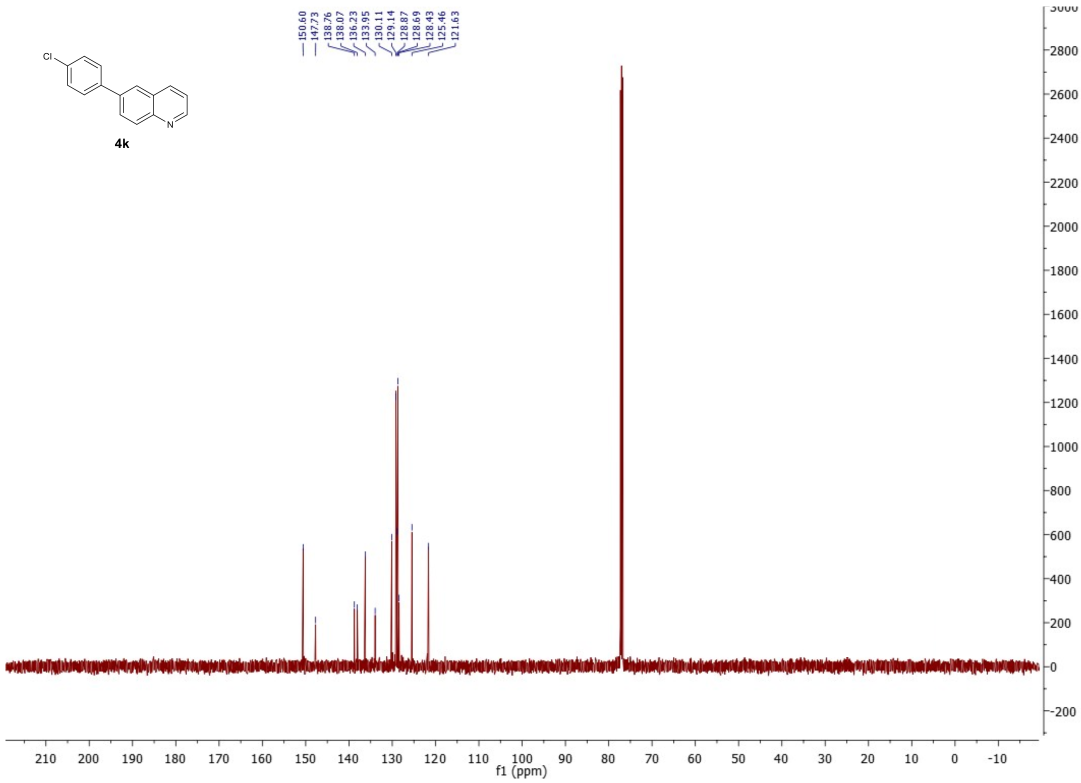
150.60
147.73
138.76
138.07
136.23
133.95
130.11
129.14
128.87
128.69
128.43
125.46
121.63
Cl
N
4k
f1 (ppm)

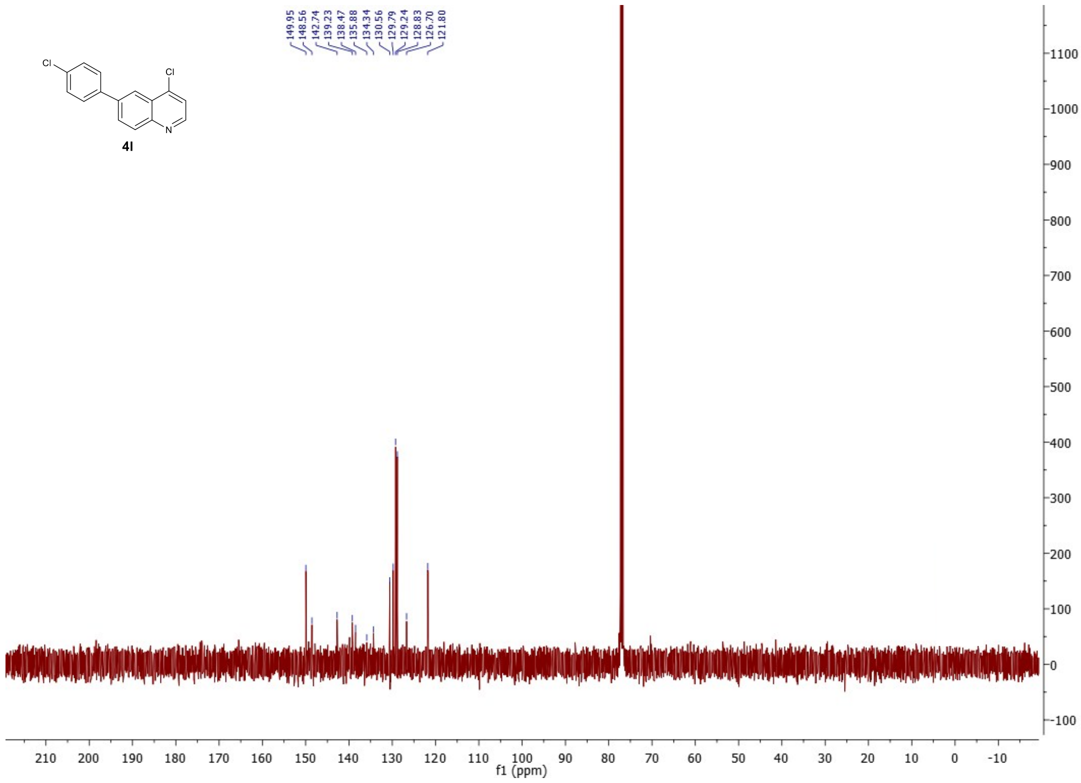
149.95
148.56
142.74
139.23
138.47
135.88
134.34
130.56
129.79
129.24
128.83
126.70
121.80
Cl
Cl
N
4l
f1 (ppm)

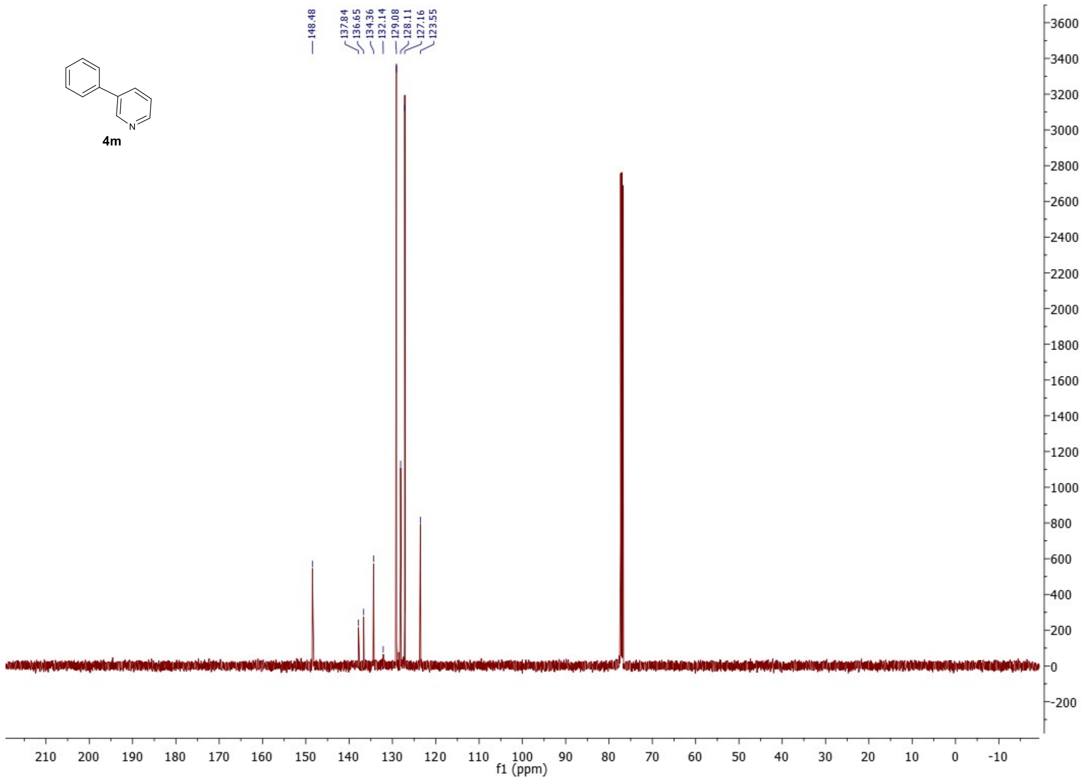

4m
148.48
137.84
136.65
134.36
132.14
129.08
128.11
127.16
123.55
f1 (ppm)

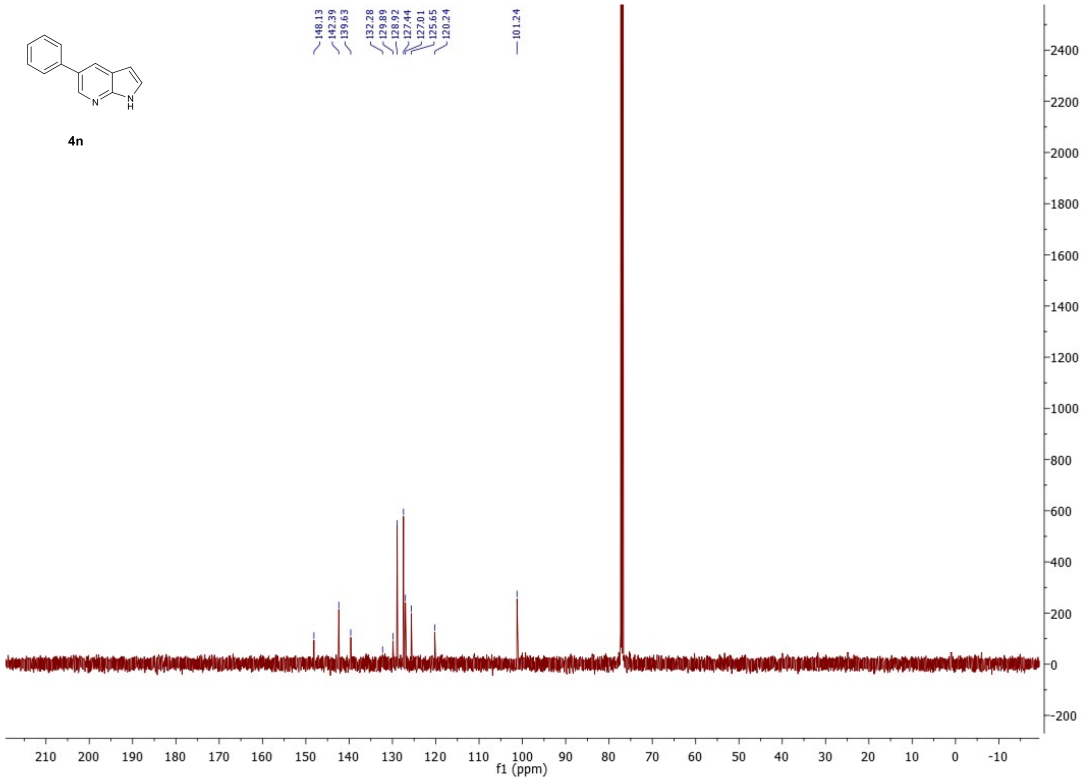

4n
148.13
142.39
139.63
132.28
129.89
128.92
127.44
127.01
125.65
120.24
101.24
f1 (ppm)

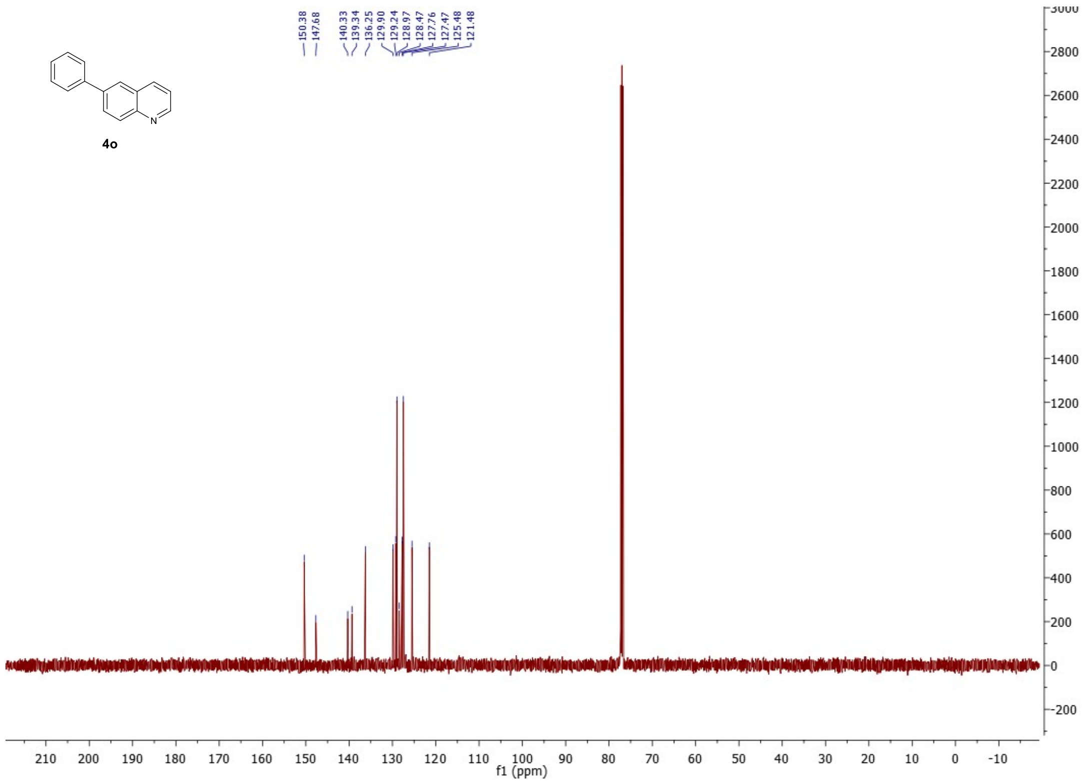
150.38
147.68
140.33
139.34
136.25
129.90
129.24
128.97
128.47
127.76
127.47
125.48
121.48
N
4o
f1 (ppm)

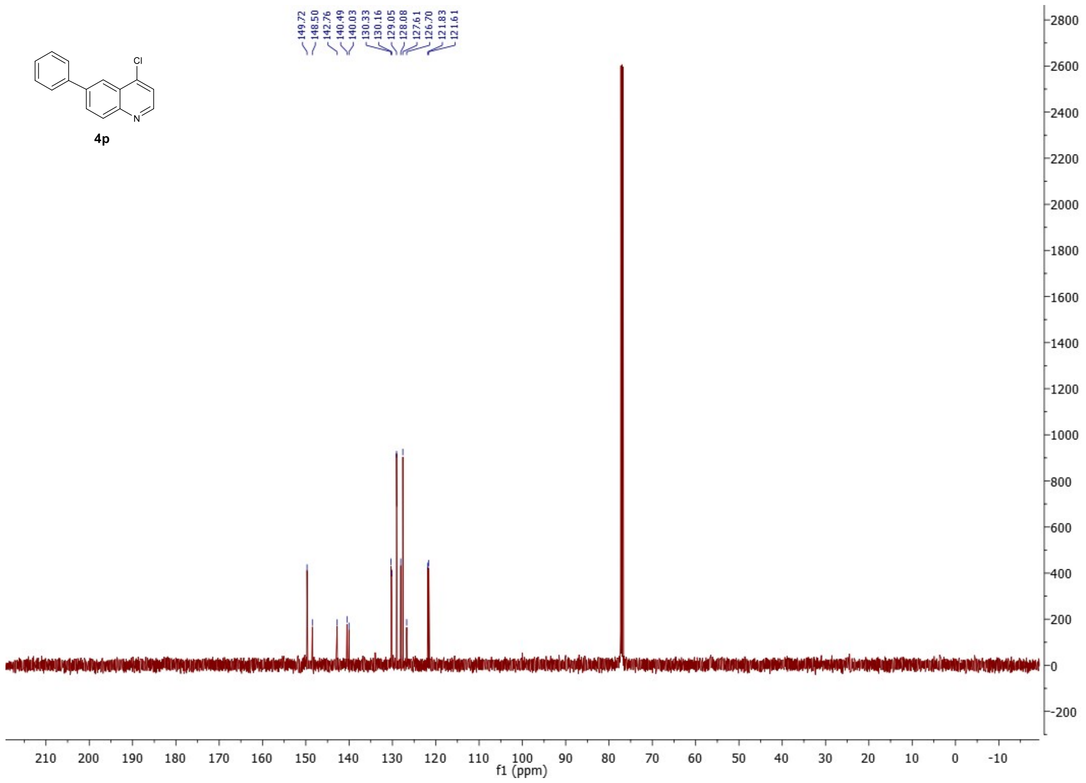
149.72
148.50
142.76
140.49
140.03
130.33
130.16
129.05
128.08
127.61
126.70
121.83
121.61
Cl
N
4p
f1 (ppm)

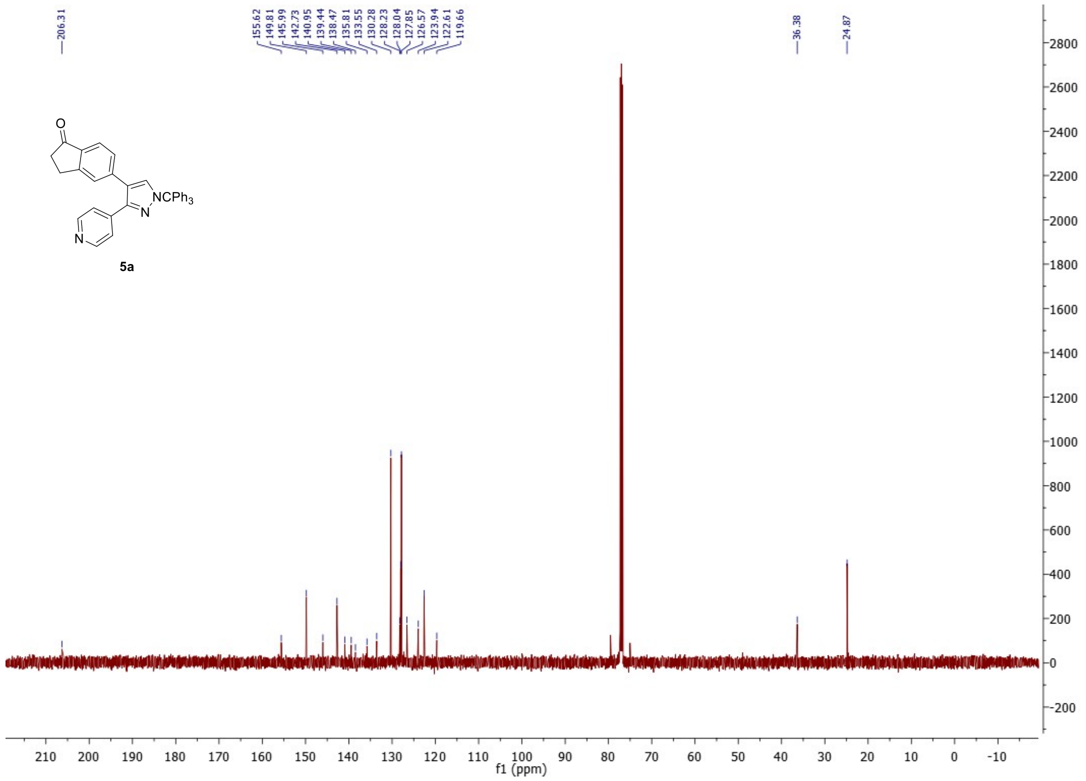
206.31
155.62
149.81
145.99
142.73
140.95
139.44
138.47
135.81
133.55
130.28
128.23
128.04
127.85
126.57
123.94
122.61
119.66
36.38
24.87
O
NCPh3
N
N
5a
f1 (ppm)

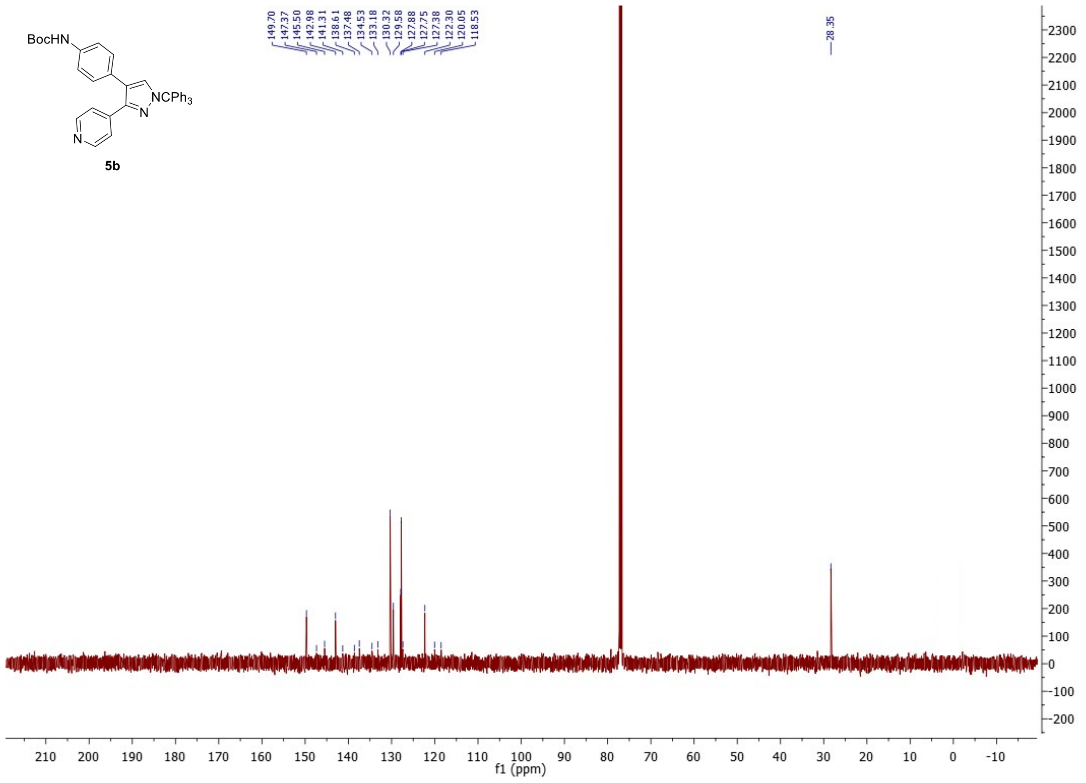
149.70
147.37
145.50
142.98
141.31
138.61
137.48
134.53
133.18
130.32
129.58
127.88
127.75
127.38
122.30
120.05
118.53
28.35
BocHN
NCPh3
N
N
5b
f1 (ppm)

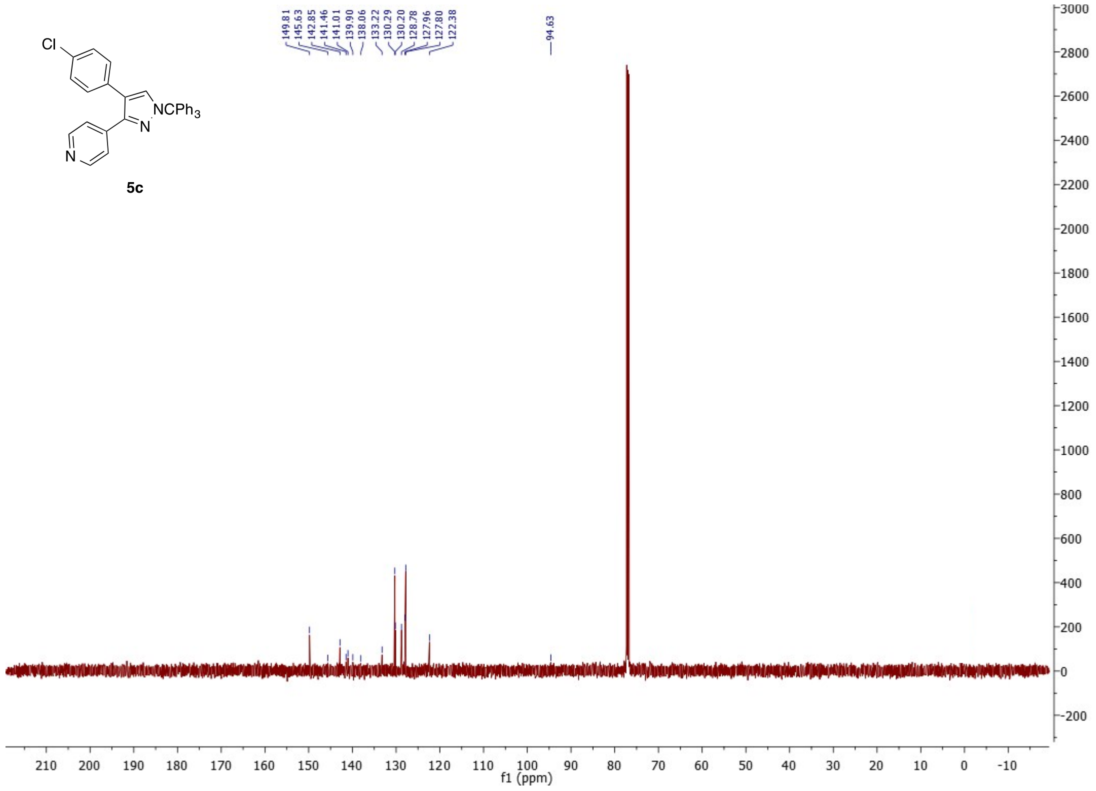
Cl
NCPh3
N
N
5c
149.81
145.63
142.85
141.46
141.01
139.90
138.06
133.22
130.29
130.20
128.78
127.96
127.80
122.38
94.63
f1 (ppm)

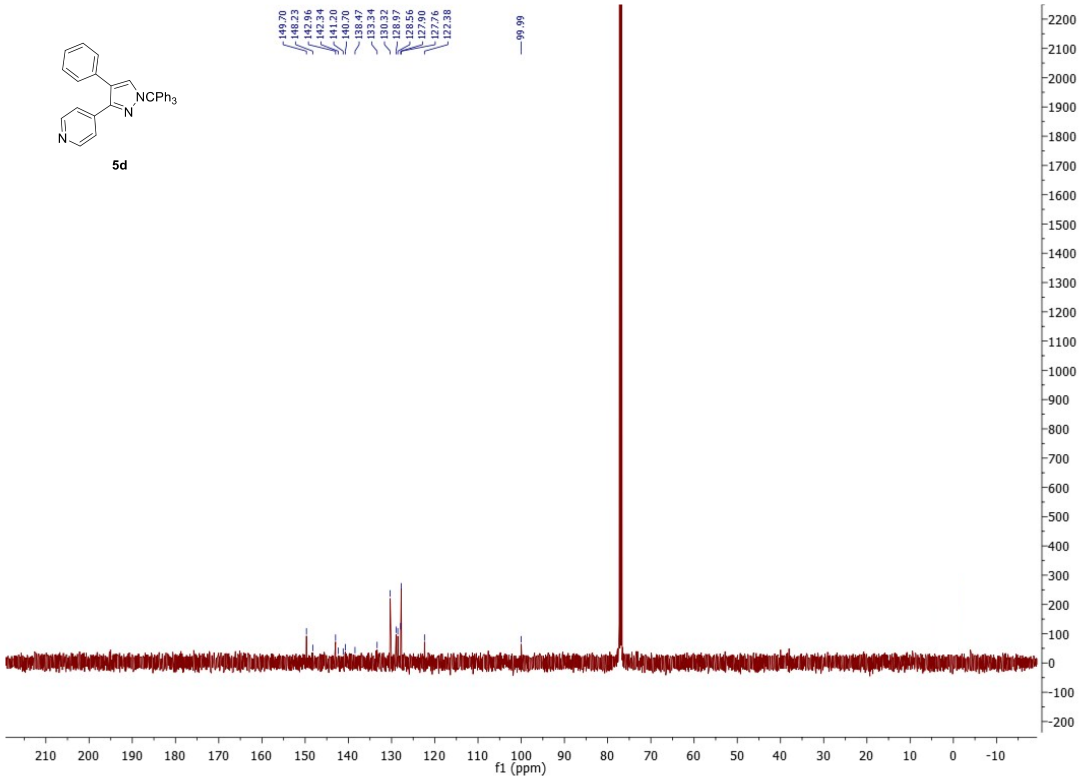
NCPh3
N
N
5d
149.70
148.23
142.96
142.34
141.20
140.70
138.47
133.34
130.32
128.97
128.56
127.90
127.76
122.38
99.99
f1 (ppm)
